# Native Top‐Down Mass Spectrometry of TAR RNA in Complexes with a Wild‐Type tat Peptide for Binding Site Mapping

**DOI:** 10.1002/anie.201610836

**Published:** 2016-12-21

**Authors:** Eva‐Maria Schneeberger, Kathrin Breuker

**Affiliations:** ^1^ Institut für Organische Chemie and Center for Molecular Biosciences Innsbruck (CMBI)Universität InnsbruckInnrain 80-826020InnsbruckAustria

**Keywords:** binding sites, collisionally activated dissociation, mass spectrometry, proteins, RNA

## Abstract

Ribonucleic acids (RNA) frequently associate with proteins in many biological processes to form more or less stable complex structures. The characterization of RNA–protein complex structures and binding interfaces by nuclear magnetic resonance (NMR) spectroscopy, X‐ray crystallography, or strategies based on chemical crosslinking, however, can be quite challenging. Herein, we have explored the use of an alternative method, native top‐down mass spectrometry (MS), for probing of complex stoichiometry and protein binding sites at the single‐residue level of RNA. Our data show that the electrostatic interactions between HIV‐1 TAR RNA and a peptide comprising the arginine‐rich binding region of tat protein are sufficiently strong in the gas phase to survive phosphodiester backbone cleavage of RNA by collisionally activated dissociation (CAD), thus allowing its use for probing tat binding sites in TAR RNA by top‐down MS. Moreover, the MS data reveal time‐dependent 1:2 and 1:1 stoichiometries of the TAR–tat complexes and suggest structural rearrangements of TAR RNA induced by binding of tat peptide.

Interactions between ribonucleic acids (RNA) and proteins are central to many fundamental biological processes, including gene expression and infection by RNA viruses. For a thorough understanding of such interactions, RNA–protein complexes are commonly investigated by nuclear magnetic resonance (NMR) spectroscopy or X‐ray crystallography, both of which require relatively large quantities of sample material. Moreover, NMR data interpretation can be complicated by unfavorable conformational dynamics,^1^ and crystallography can become impossible if a complex fails to crystallize properly. Strategies based on (photo)chemical crosslinking^2^ have the advantage that they can be performed in vivo^3^ but can variously suffer from low crosslinking yields, different crosslinking reactivity of different residues, or the formation of intramolecular instead of intermolecular crosslinks.^4^ Moreover, crosslinking reagents generally target specific functional groups such as amines or thiols that may not be present in a binding region, and efficient crosslinking can require pH values that may not be compatible with RNA–protein complex stability.^5^ Although all of the above techniques can provide highly important structural data, each of them requires laborious sample preparation procedures, that is, crystallization, the introduction of heavy isotopes, or optimization of the reaction conditions for the formation of intermolecular crosslinks.

As an alternative to these methods, we explore the potential of native top‐down mass spectrometry (MS) using electrospray ionization (ESI)^6^ for the characterization of RNA–protein interactions. Previous studies showed that native ESI can produce gaseous RNA–protein or RNA–ligand complexes,^7^ and in top‐down MS experiments using collisionally activated dissociation (CAD), Loo^8^ and Fabris7f observed cleavage of covalent mononucleotide phosphate and RNA phosphodiester bonds, respectively, rather than dissociation of the noncovalent bonds of the complexes studied. In agreement with our recent work on KIX protein^9^ and data from the literature,^10^ their findings suggest that in the absence of solvent, the strength of salt bridges can be comparable to that of covalent bonds. By contrast, desolvated duplexes of small interfering RNA that are stabilized by base pairing and stacking instead of salt‐bridge interactions were found to dissociate by strand separation rather than backbone bond cleavage.^11^ Coincidentally, electrostatic interactions are common elements of RNA–protein binding; a statistical analysis of crystal structures revealed a high percentage of positively charged amino acid residues in the binding interfaces with the negatively charged RNA.^12^

As a model system for our studies, we investigated complexes of 31 nt TAR (transactivation responsive) RNA7a (Scheme 1 a) and a basic peptide (H_2_N‐GRKKRRQRRRPP‐NH_2_) comprising the arginine‐rich binding region of tat (trans‐activating) protein from human immunodeficiency virus type 1 (HIV‐1). Previous studies showed that MS can be used to probe the effect of relative TAR and tat concentrations on complex stoichiometry.7a, 13 Solution NMR studies of the HIV‐1 TAR–tat complex were generally complicated by broad signals,^14^ but highly converging structures of complexes with peptide mimetics of tat were obtained by Varani and co‐workers.^15^

**Scheme 1:**
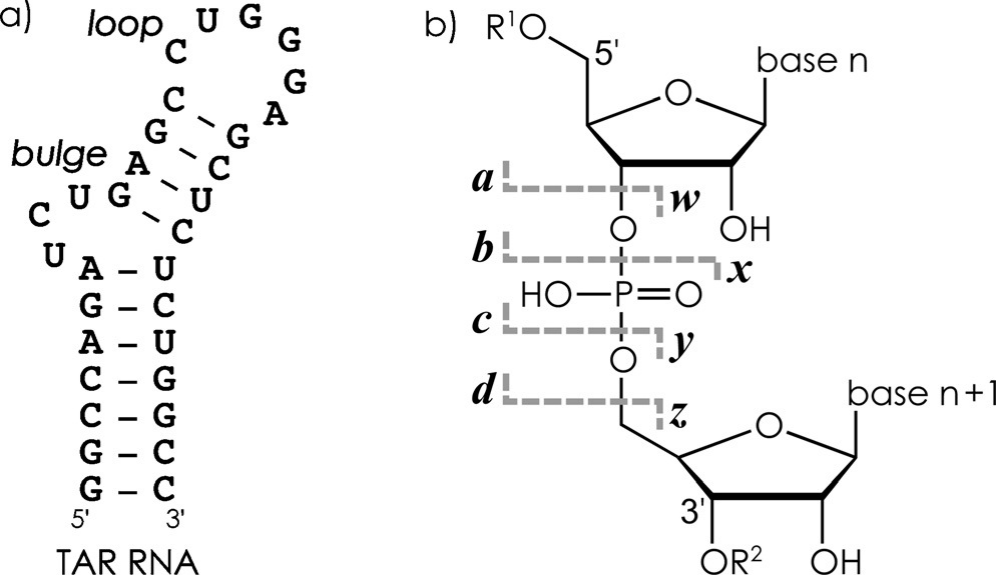
a) Predicted secondary structure (http://rna.tbi.univie.ac.at)^16^ of the TAR RNA construct studied. b) Nomenclature of fragment ions from RNA backbone cleavage.

ESI approximately 5 min after the preparation of a 1:1 TAR/tat solution produced 24 % unbound RNA, that is, (TAR−nH)^n−^, 42 % 1:1 complex, (TAR+tat−nH)^*n*−^, and 34 % 1:2 complex, (TAR+2tat−nH)^n−^ (these proportions changed somewhat with pH, see Figure S1 in the Supporting Information), but ESI after approximately 30 min produced mostly the 1:1 complex (Figure 1 a and the Supporting Information, Figure S1). This data illustrates that native top‐down MS can provide time‐resolved information on complex assembly and stoichiometry7a, 13 that is not generally available by other methods.

**Figure 1:**
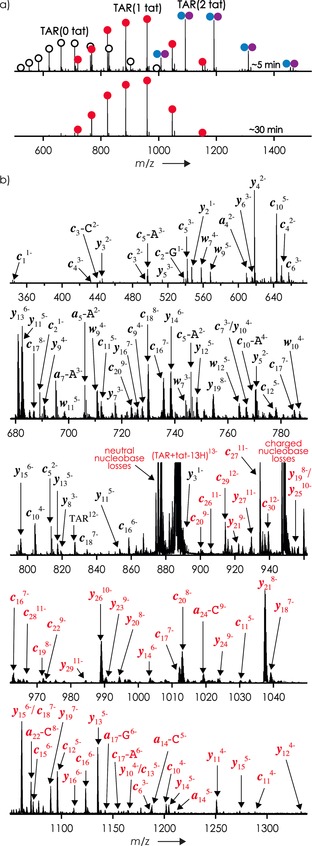
a) ESI spectra of TAR and tat (2 μm each) in 9:1 H_2_O/CH_3_OH at pH 7.7 (adjusted by addition of piperidine, final concentration 1.125 mm) sprayed ca. 5 (top) and 30 min (bottom) after preparation of the solution. Empty circles, (TAR−nH)^*n*−^; red circles, (TAR+tat−nH)^*n*−^; blue/purple circles, (TAR+2 tat−nH)^*n*−^ ions. b) CAD spectrum of (TAR+tat−13H)^13−^ ions at 120.9 eV laboratory frame energy (36 % dissociation), TAR fragments with (red) and without (black) tat attached are labeled.

CAD of (TAR+tat−13H)^13−^ ions (*m*/*z* ca. 886; Figure 1 b) produced only 0.3 % unbound TAR RNA and 0.2 % free tat peptide but 26.1 % products from RNA backbone cleavage (Scheme 1 b), that is, ***c*** (9.6 %), ***y*** (12.0 %), ***a*** (1.8 %), ***w*** (2.4 %), and ***i*** (internal) fragments from secondary backbone cleavage (0.3 %), along with 64.4 % undissociated ions and 9.0 % nucleobase loss from the latter. Nucleobase loss from fragments was also observed in 17.2 %, 5.1 %, 72.7 %, and 0.4 % of all ***c***, ***y***, ***a***, and ***w*** ions, respectively; the substantially higher value for ***a*** ions is consistent with the dominant mechanism proposed for 3′ C−O cleavage that is initiated by nucleobase loss.^17^ The lower‐energy mechanism of phosphodiester backbone bond cleavage into ***c*** and ***y*** ions (Supporting Information, Figure S2), on the other hand, does not involve nucleobase loss.^18^ Site‐specific yields of ***c*** and ***y*** ions were generally higher on the 5′‐side of guanosine (Figure 2 a), which we have recently attributed to the hydrogen bonding between nucleobase and phosphodiester moieties at the cleavage site prior to dissociation.^18^ Peptide binding somewhat reduces this effect (Supporting Information, Figure S3), consistent with the restricted conformational RNA flexibility in the bound state.

**Figure 2:**
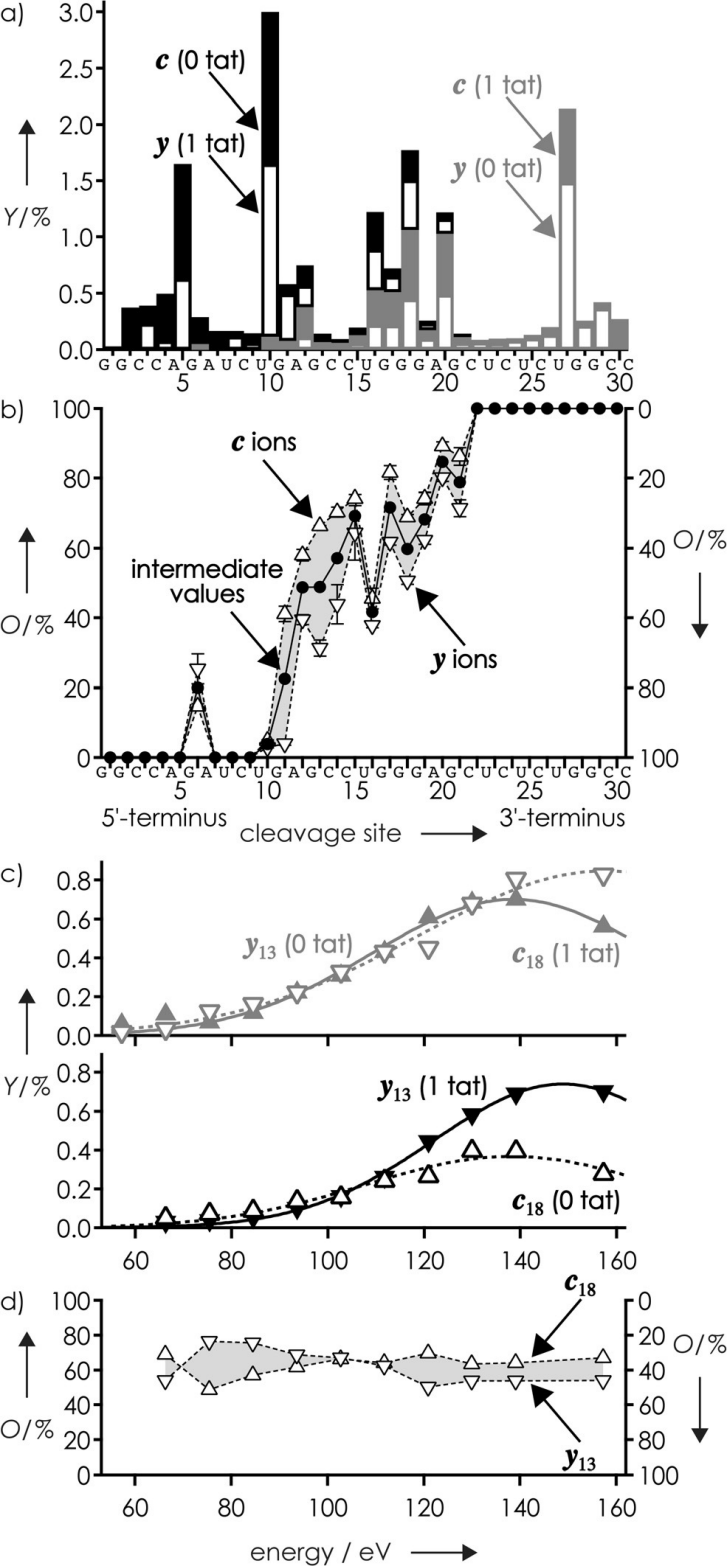
For CAD of (TAR+tat−13H)^13−^ ions, a) yield (*Y*) of ***c*** and ***y*** fragments without (0 tat) and with (1 tat) peptide attached from spectrum in Figure 1 and b) average level of tat occupancy (*O*) of ***c*** (▵, left axis) and ***y*** (▿, right axis) fragments (from triplicate measurements under identical experimental conditions at 120.9 eV, error bars show standard deviation of the mean) and intermediate values (•, calculated as (*O*(***c***)−*O*(***y***)+100)/2) versus cleavage site. c) Yield of complementary ***c***_18_(1 tat) and ***y***_13_(0 tat) fragments (top) and complementary ***c***_18_(0 tat) and ***y***_13_(1 tat) fragments (bottom) and d) occupancy values for ***c***_18_ and ***y***_13_ versus energy used for CAD; lines are meant to guide the eye.

Moreover, the CAD spectrum in Figure 1 b shows ***c***, ***y***, ***a***, and ***w*** fragments from RNA backbone cleavage both with and without the intact peptide attached, the *m*/*z* values of which were consistently higher with the peptide attached than without, regardless of the fragment ion mass (Supporting Information, Figure S4). This suggests that some of the net positive charge of the tat peptide is preserved in both the (TAR+tat−13H)^13−^ ions and its ***c***, ***y***, ***a***, and ***w*** fragments and corroborates the hypothesis that electrostatic interactions play a major role in stabilizing the gaseous TAR–tat complex. Importantly, noncovalent bonds between TAR and tat as well as the covalent bonds of tat can apparently be preserved at energies that are sufficiently high to cleave covalent RNA‐backbone bonds and disrupt intramolecular RNA interactions such as base pairing in stem regions (Scheme 1 a).

Figure 2 b illustrates the level of tat occupancy of the ***c*** and ***y*** fragments of TAR RNA for each cleavage site, calculated as 100⋅*Y*(***c*** or ***y*** with tat)/(*Y*(***c*** or ***y*** with tat)+*Y*(***c*** or ***y*** without tat)); within error limits, these were the same for ESI circa 5 and 30 min after preparation of the solution. Zero occupancy was found for ***c***_1_–***c***_5_, ***c***_7_–***c***_9_, ***y***_2_–***y***_9_ (***y***_1_ was not detected) and 100 % occupancy for the complementary ***y***_30_–***y***_26_, ***y***_24_–***y***_22_, ***c***_30_–***c***_22_ fragments, suggesting that TAR residues 1–5, 7–9, and 22–31 did not, or at least not strongly, interact with tat. Occupancy values other than 0 % or 100 % were found for all other sites, for example, from cleavage at site 18, 68.9±0.8 % for ***c***_18_ and 49.4±1.0 % for its complement ***y***_13_. This can be rationalized by the interactions of tat peptide with more than one residue of TAR RNA and the breaking by CAD of only some of them according to the strength and number of interactions with each fragment. In the above example, the higher fraction of ***c***_18_ fragments with tat peptide attached compared to that of ***y***_13_ suggests an overall higher stability of tat interactions with ***c***_18_ than with ***y***_13_, but both are sufficiently strong to prevent loss of tat during the backbone cleavage of TAR.

The added occupancy values of complementary ***c*** and ***y*** fragments, however, generally exceeded or fell below the expected 100 %; for cleavage at site 18, the values for ***c***_18_ and ***y***_13_ add up to 118 %. The discrepancies of the added occupancy values from 100 % ranged from 2 % at site 10 to 37 % at site 11, with an average value of 17 %, and can be attributed to different stabilities of the ***c*** and ***y*** fragments, with and without peptide attached, against secondary backbone cleavage. To exemplify this point, yields of complementary ***c***_18_(1 tat) and ***y***_13_(0 tat) fragments (top) and ***c***_18_(0 tat) and ***y***_13_(1 tat) fragments (bottom) are illustrated in Figure 2 c as a function of the energy used for CAD. Apparently, ***c***_18_ fragments undergo secondary backbone cleavage more readily than ***y***_13_ fragments, and ***c***_18_(1 tat) showed higher stability than ***c***_18_(0 tat). Because secondary backbone cleavage of a ***c*** or ***y*** fragment produces both an internal ***i*** fragment and a smaller ***c*** or ***y*** fragment, it not only decreases the yield of larger ***c*** or ***y*** fragments (Figure 2 c) but also increases the yield of smaller ***c*** or ***y*** fragments (Supporting Information, Figure S5).^19^ For the determination of the occupancy values, spectra obtained at lower CAD energy (at which fewer products from secondary backbone cleavage are observed)^19^ should thus be more accurate; on the other hand, both the signal‐to‐noise level and sequence coverage generally increase with increasing energy (Supporting Information, Figure S2). Full sequence coverage in CAD of (TAR+tat−13H)^13−^ ions was obtained at energies greater than or equal to 111.8 eV, and the yields of the internal fragments were less than 1 % at energies below 130 eV, with 120.9 eV (Figure 1 b, Figure 2 a,b) just in between.

The occupancy values of ***c*** and ***y*** fragments increased almost monotonically with increasing number of residues (Figure 2 b); however, some variation outside of the error limits, even when only intermediate values were considered, was nonetheless observed. For example, the tat occupancy of ***c***_6_ was circa 15 %, but 0 % for ***c***_7_, ***c***_8_, and ***c***_9_, and that of the complementary fragments circa 75 % for ***y***_25_ but 100 % for ***y***_24_, ***y***_23_, and ***y***_22_; likewise, the occupancy values at site 16 stand out. The variations in occupancy values, however, do not appear to be related to the net charge of the fragment ions, as these showed a steady increase with increasing number of residues (Supporting Information, Figure S4). We instead attribute this phenomenon to charge redistribution within fragment ions by proton transfer according to Coulombic repulsion,^18^ after backbone cleavage but before complex dissociation, that can weaken or strengthen individual interactions differently in different fragment ions, which in turn affects the competition between complementary ***c*** and ***y*** fragments for tat. In this scenario, an electrostatic interaction of residue 6 of ***c***_6_ with tat can be sufficiently strong to compete with the interactions between the complementary fragment ***y***_25_ and tat, whereas in the ***c***_7_, ***c***_8_, and ***c***_9_ ions, negative charge will move away from residue 6 to the terminal residues 7, 8, and 9, respectively,^18^ thus weakening the electrostatic interaction of residue 6 with tat. This does not exclude the possibility of tat interactions with residues 7, 8, and 9 but suggests that they must be substantially weaker than that between tat and residue 6 of ***c***_6_.

Nevertheless, the data in Figure 2 b clearly show that in the 1:1 TAR–tat complex, tat binds to residues 6 and 10–22 of TAR RNA. The color coding of residues 6 and 9–22 (red) and the more weakly interacting residue 10 grouped with the possibly weakly interacting residues 7–9 (orange) of the ground state structure of TAR in complex with a peptide mimetic (green)15b shows excellent agreement between MS and NMR data (Figure 3 a), even though the tat peptide studied here was linear and that in the NMR structure was cyclic. In the same manner, data from CAD MS of (TAR+2 tat−12 H)^12−^ ions (Supporting Information, Figures S6 and S7) revealed the sites of tat binding to TAR in the 1:2 TAR–tat complex (Figure 3 b). In the 1:1 complex, tat binds to both the predicted loop, bulge, and upper 5′‐stem region, whereas in the 1:2 complex, one tat binds to the loop and upper 3′‐stem region (blue), and the other to the bulge and 5′‐stem region (violet). Consistent with the rather ill‐defined NMR structure of free TAR,^20^ our data suggest a dynamic and sufficiently open TAR RNA structure to which two tat peptides can initially bind, followed by rearrangements into a more compact TAR structure along with ejection of one peptide.

**Figure 3:**
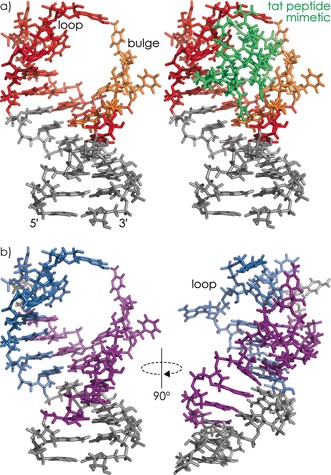
NMR ground state structure of TAR RNA in complex with peptide mimetic (PDB entry 5J0M)15b with tat binding sites from MS data color‐coded (see main text) for a) 1:1 and b) 1:2 TAR–tat complex.

In conclusion, we have shown that native top‐down MS provides time‐resolved information on TAR–tat complex stoichiometry and reveals tat binding sites at the single‐residue level of TAR RNA. Apparently, the electrostatic interactions between TAR RNA and the basic tat peptide in the gas phase are sufficiently strong to survive at CAD energies that cleave phosphodiester backbone bonds of RNA. Native top‐down MS requires only small amounts of sample without the need for laborious preparation procedures, allows for the separate study of species that differ in *m*/*z*, simultaneously provides extensive sequence information (Supporting Information, Tables S1 and S2),^21^ and is a promising approach for probing RNA–protein binding, especially in cases in which conventional methods for structural probing are not applicable.

## Experimental Section

Experiments were performed on a 7 T Fourier transform ion cyclotron resonance (FT‐ICR) mass spectrometer (Bruker, Austria) equipped with an ESI source, a linear quadrupole for ion isolation, and a collision cell for CAD.

## Conflict of interest

The authors declare no conflict of interest.

## Supporting information

As a service to our authors and readers, this journal provides supporting information supplied by the authors. Such materials are peer reviewed and may be re‐organized for online delivery, but are not copy‐edited or typeset. Technical support issues arising from supporting information (other than missing files) should be addressed to the authors.

Supplementary
