# Supplementary material for: Native Top‐Down Mass Spectrometry of TAR RNA in Complexes with a Wild‐Type tat Peptide for Binding Site Mapping
Source: Angew Chem Int Ed Engl. 2016 Dec 21;56(5):1254–8. doi: 10.1002/anie.201610836 (PMC5299493; doi:10.1002/anie.201610836)
Supplement: Supplementary file 1 — Supplementary [file ANIE-56-1254-s001.pdf]

Angewandte Chemie
Eine Zeitschrift der Gesellschaft Deutscher Chemiker

Supporting Information

Native Top-Down Mass Spectrometry of TAR RNA in Complexes with a Wild-Type tat Peptide for Binding Site Mapping

*Eva-Maria Schneeberger and Kathrin Breuker**

anie_201610836_sm_miscellaneous_information.pdf

Supporting Information

Sample preparation for ESI of TAR-tat complexes

TAR RNA was custom synthesized (Dharmacon, Austria), H_2O was purified to 18 MΩ·cm at room temperature using a Milli-Q system (Millipore, Austria), CH_3OH (Acros, Austria) was HPLC-grade, and ammonium acetate ("for mass spectrometry") and piperidine (>99.5%) were purchased from Sigma-Aldrich (Austria). For desalting, 500 μl RNA solution (up to 20 μM) was concentrated to 100 μl using Vivaspin 500 centrifugal concentrators (Sartorius, Austria, PES membrane, MWCO 5000), and 400 μl ammonium acetate solution (100 mM) was added. The process was repeated five times, followed by six cycles of concentration and dilution with H_2O. The 12-residue tat peptide (residues 48-57 of tat protein) was purchased from Sigma-Aldrich (Austria), and used without further purification.

Prior to each experiment, desalted TAR RNA solution (10-15 μM in H_2O) was heated to 90 °C for 90 s, followed by cooling on ice for 10 min and thermal equilibration for another 10 min at room temperature. Solutions for ESI (flow rate 1.5 μl/min) were prepared by diluting TAR RNA and tat peptide stock solutions to a final concentration of 2 μM each in 9:1 vol H_2O/CH_3OH with piperidine as additive (750 μM - 2 mM). Between 10 and 50 (ESI) or 100 and 500 (CAD) scans were added for each spectrum, and data reduction utilized the SNAP2 algorithm (Bruker, Austria). Mass values of molecular (ESI) or fragment (CAD) ions without peptide attached were used for internal calibration of the spectra (standard deviation <0.5 ppm, see Tables S1 and S2).

Time-resolved ESI MS of TAR-tat complexes

For time-resolved experiments (Figure S1c), ESI was operated in either "offline" or "online" mode. In offline-ESI experiments, solution was prepared, loaded into a syringe that was connected by PEEK tubing to the sample inlet of the ESI-source (Apollo II, Bruker), and the spray started by turning on the syringe pump at a flow rate of 1.5 μl/min. Up to 4 spectra were recorded in less than 4 minutes before disconnecting the syringe and thoroughly rinsing all parts with solvent (9:1 H_2O/CH_3OH at pH 7.7, adjusted by addition of piperidine). For online-ESI, solution was prepared, loaded into the syringe, and continuously sprayed for up to 80 min, during which spectra were recorded in 5 min intervals.

Calculation of fragment yields

Fragment yields were calculated as %-values of the sum of all ion abundances, considering that RNA backbone cleavage gives a pair of complementary $\boldsymbol{c}$ and $\boldsymbol{y}$ or $\boldsymbol{a}$ and $\boldsymbol{w}$ ions, and taking into account that the formation of internal fragments requires two backbone cleavages: 100% = 0.5·[c] + 0.5·[y] + 0.5·[a] + 0.5·[w] + 0.33·[i] +0.5·[TAR] + 0.5·[tat] + [molecular ions and loss of small neutral species from the latter], with $\boldsymbol{c}$, $\boldsymbol{y}$, $\boldsymbol{a}$, ·$\boldsymbol{w}$, ·and $\boldsymbol{i}$ ion concentrations including base losses.

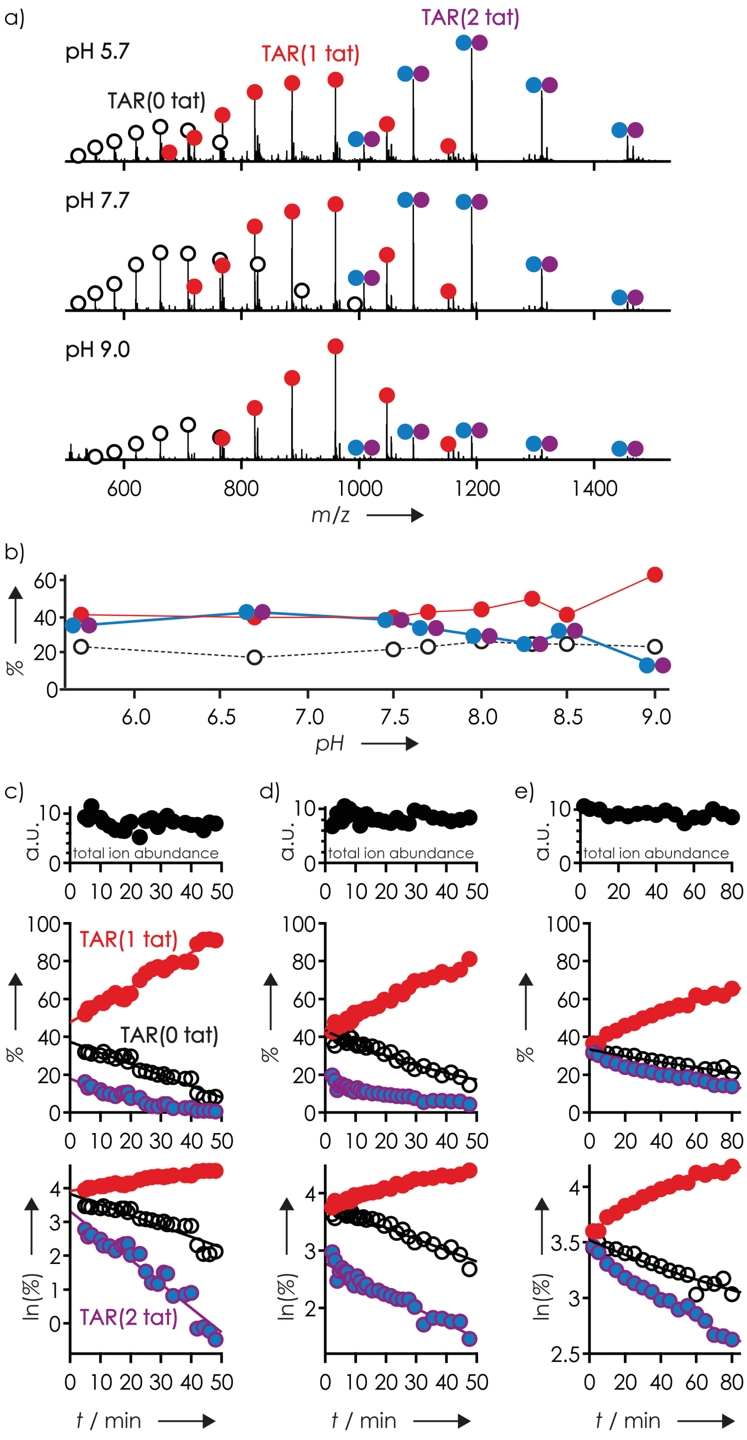

Figure S1. ESI of TAR and tat (2 μM each) in 9:1 H_2O/CH_3OH ~5 min after preparation of the solutions, a) at pH 5.7, 7.7, and 9.0 as indicated (adjusted by addition of piperidine), and b) proportion of O: $(TAR\text{-}nH)^{n-}$, ●: $(TAR+tat\text{-}nH)^{n-}$, ●●: $(TAR+2tat\text{-}nH)^{n-}$ ions versus solution pH; c-e) for ESI at pH 7.7, total ion abundance (top, in arbitrary units, a.u.) and proportion of $(TAR\text{-}nH)^{n-}$, $(TAR+tat\text{-}nH)^{n-}$, and $(TAR+2tat\text{-}nH)^{n-}$ ions on linear (middle) and logarithmic (bottom) scales versus time after preparation of the solutions, monitored by c) offline-ESI and d,e) online ESI, both of which produced stable total ion abundances (top). The slower kinetics observed with online-ESI (d,e) indicate substantial sample carry-over, however, in all experiments, the proportion of $(TAR+2tat\text{-}nH)^{n-}$ ions steadily decreased over time in favor of $(TAR+tat\text{-}nH)^{n-}$ ions, consistent with our hypothesis that TAR can initially bind two tat peptides, one of which is eventually ejected.

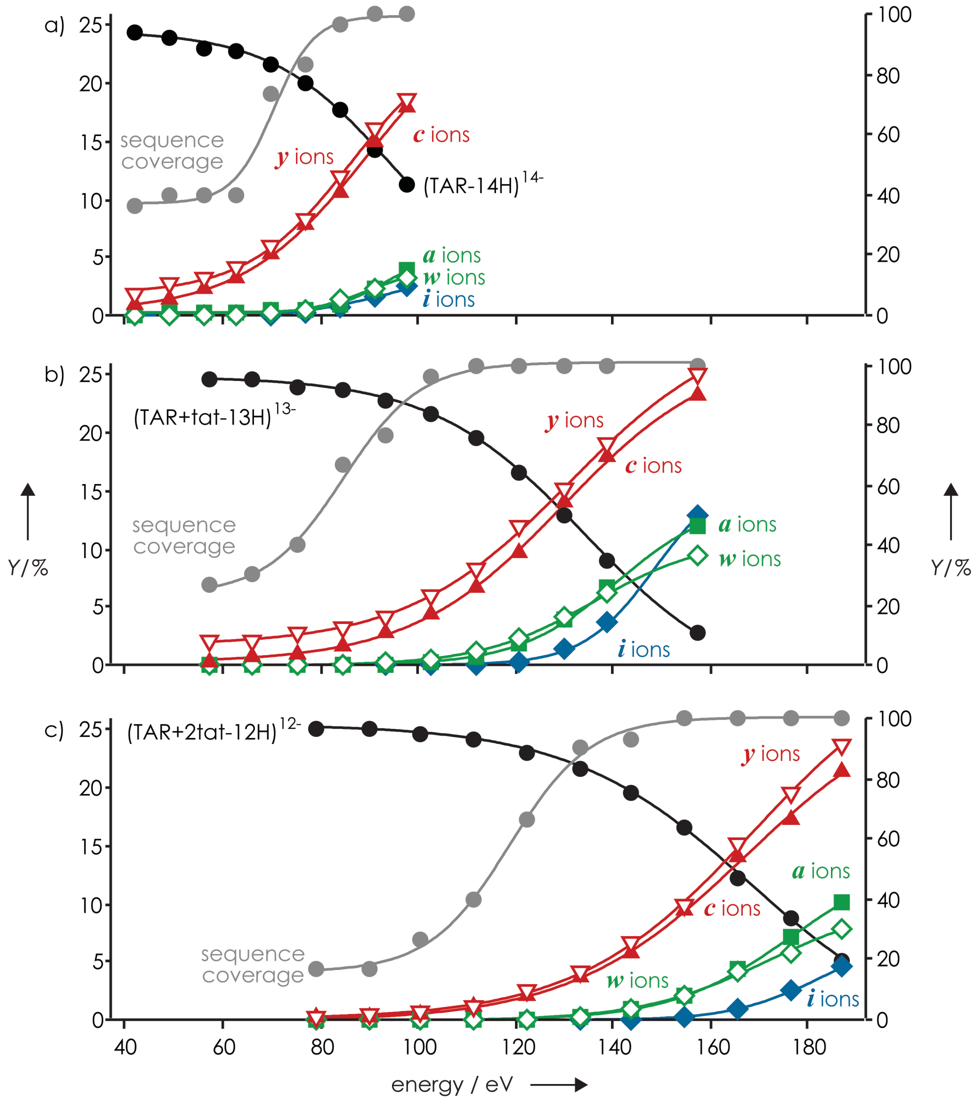

Figure S2. Yield of ***c***, ***y***, ***a***, ***w***, and ***i*** fragments (left axis) and yield of undissociated ions and sequence coverage (right axis) from CAD of a) $(TAR\text{-}14H)^{14-}$, b) $(TAR+tat\text{-}13H)^{13-}$, c) $(TAR+2tat\text{-}12H)^{12-}$ ions versus laboratory frame energy (bottom axis); lines are meant to guide the eye. Three major factors contribute to differences in energy required for dissociation of $(TAR\text{-}14H)^{14-}$, $(TAR+tat\text{-}13H)^{13-}$, and $(TAR+2tat\text{-}12H)^{12-}$ ions. First, each tat added to TAR increases the number of vibrational degrees of freedom over which energy is distributed, second, a decrease in ion net charge generally increases the energy required for dissociation, and third, ion structure and intramolecular charge distribution play a role. Because the exact contributions of these factors cannot be quantified, we used energies that produced the same extent of dissociation (36%) for assignment of binding sites in 1:1 and 1:2 TAR-tat complexes in Figure 3.

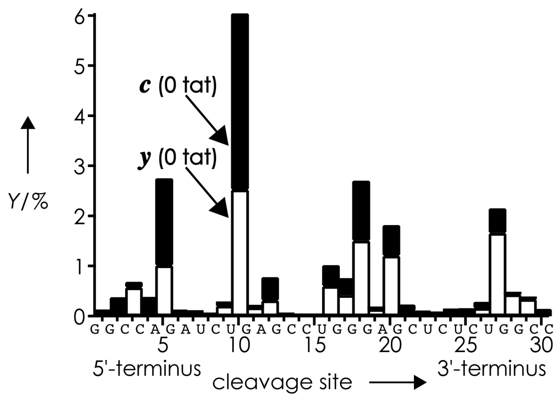

Figure S3. Yield of *c* and *y* fragments from CAD of (TAR-14H)[14-] ions (without tat peptide attached, 0 tat) at 84 eV laboratory frame energy (32% dissociation).

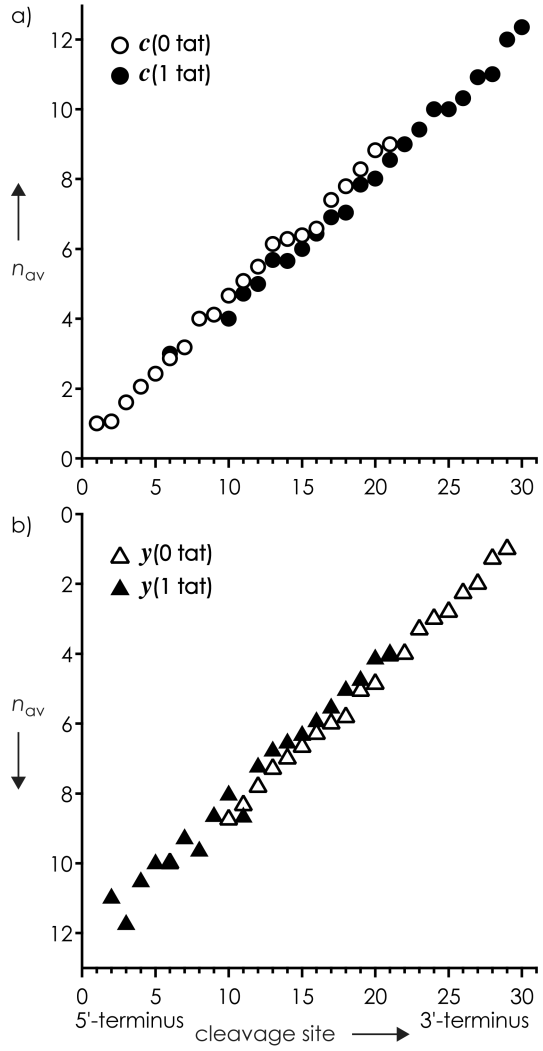

Figure S4. Average charge n_{av} of a) *c* and b) *y* fragments with and without tat from CAD of (TAR+tat-13H)[13-] ions at 120.9 eV laboratory frame energy versus cleavage site.

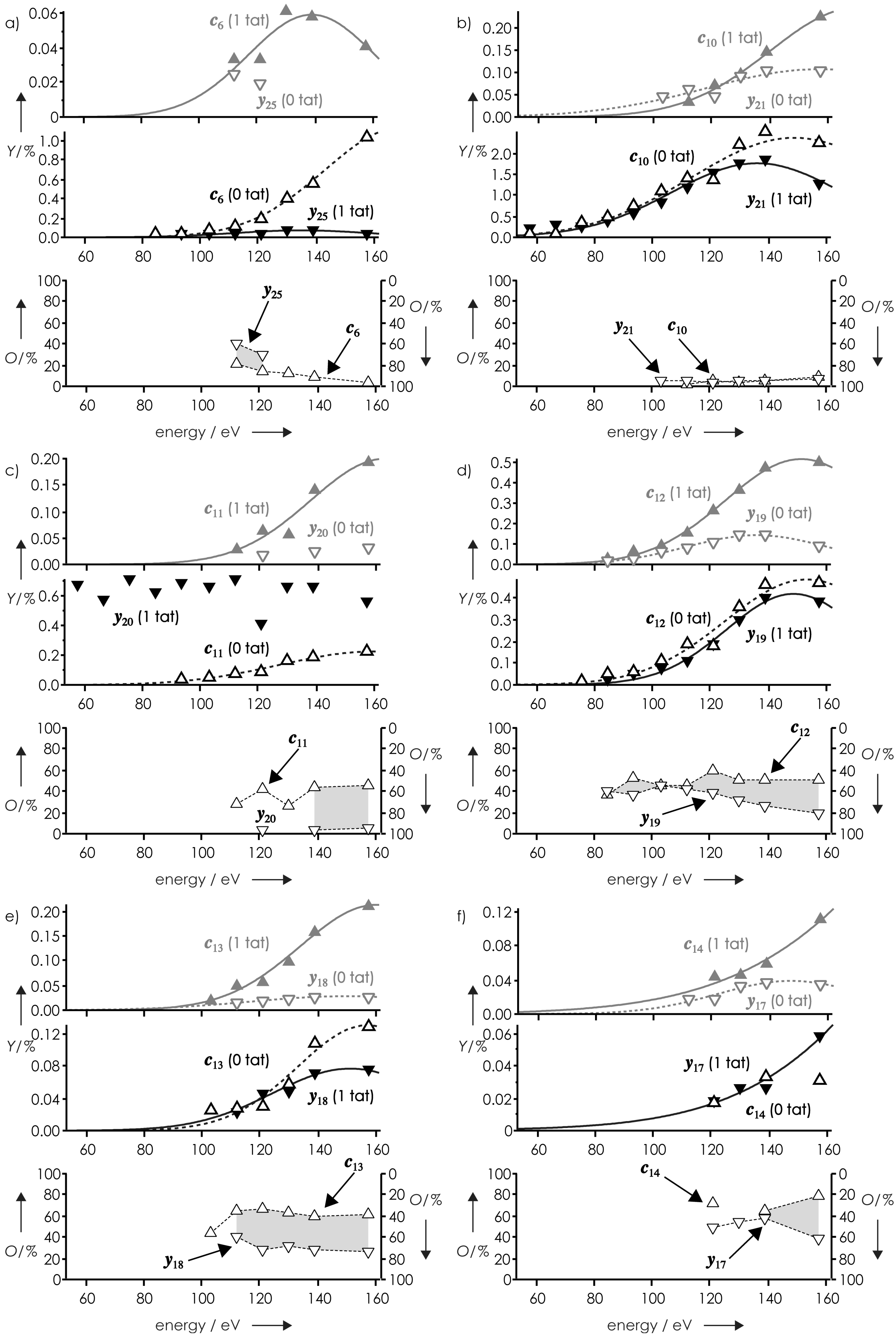

Figure S5. As in Figures 2c and 2d, yield of c and y ions from CAD of (TAR+tat-13H)$^{13-}$, and level of occupancy with tat peptide vs. laboratory frame energy for cleavage at sites 6 (a) and 10-14 (b-f).

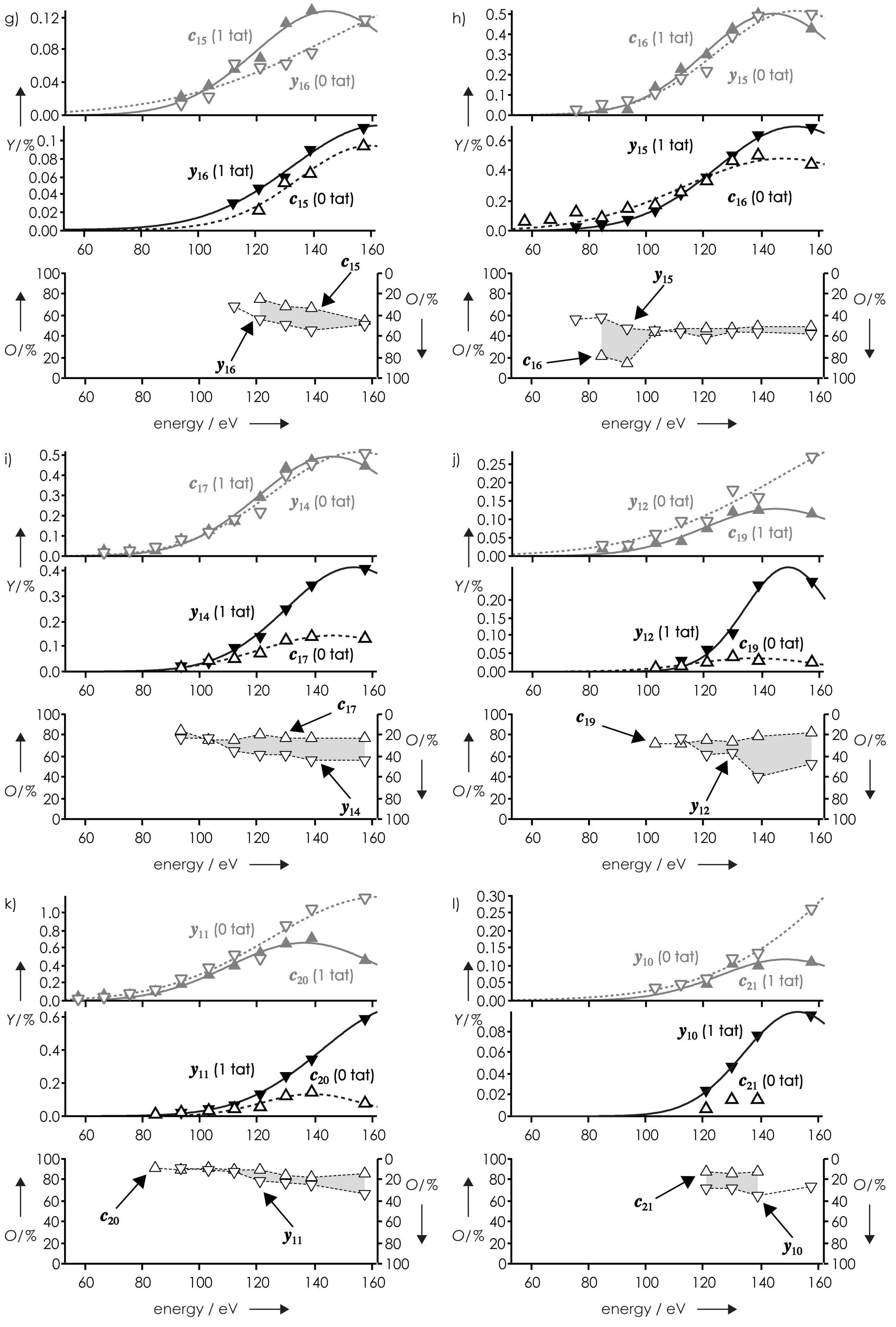

Figure S5 continued. yield of c and y ions from CAD of $(TAR+tat-13H)^{13-}$, and level of occupancy with tat peptide vs. laboratory frame energy for cleavage at sites 15-17 (g-i) and 19-21 (j-l).

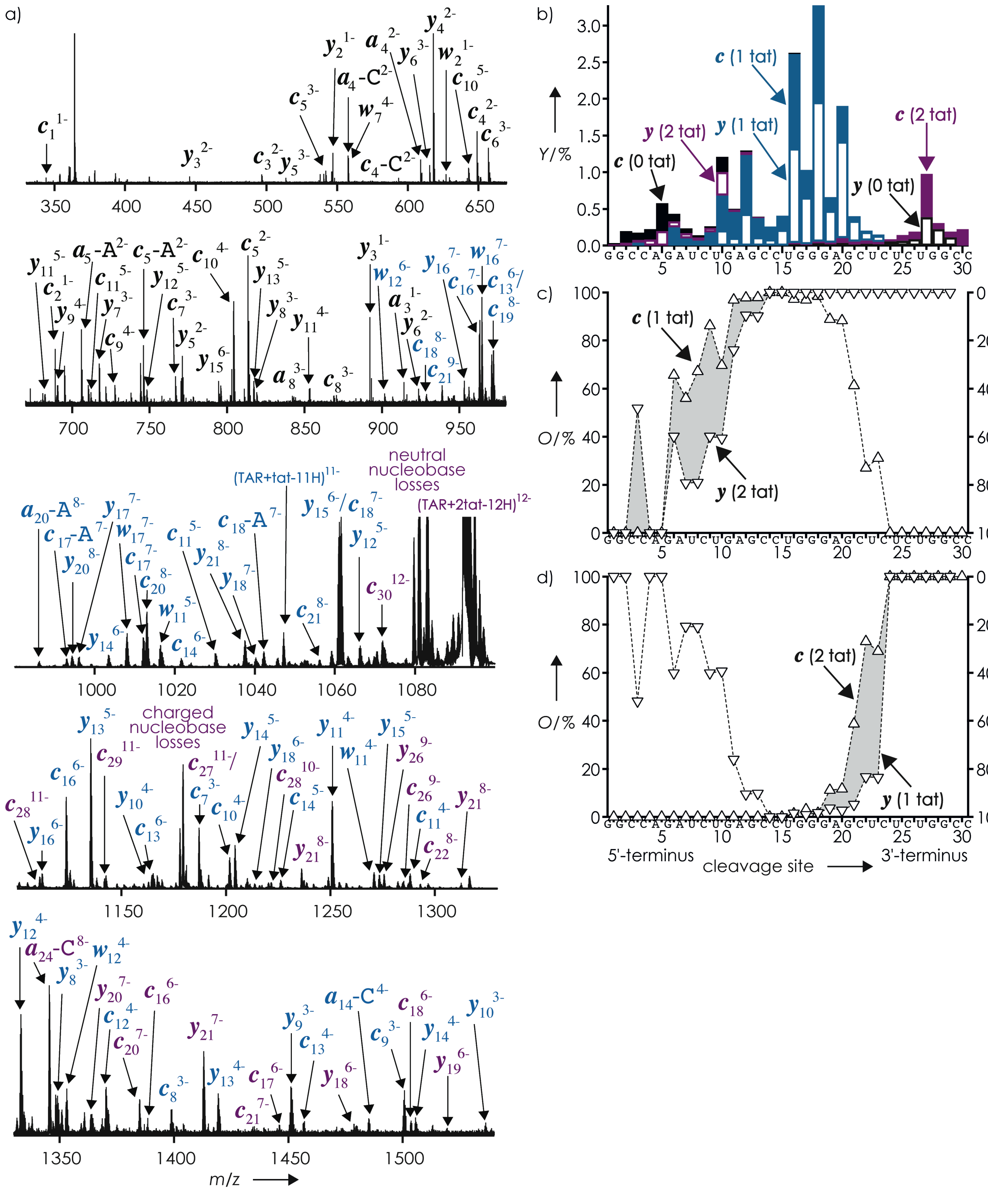

Figure S6. a) CAD spectrum of (TAR+2tat-12H)$^{12-}$ ions at 154.8 eV laboratory frame energy (36% dissociation), with ***c***(0 tat) and ***y***(0 tat) fragments labeled in black, ***c***(1 tat) and ***y***(1 tat) in blue, and ***c***(2 tat) and ***y***(2 tat) in violet; b) yield of ***c*** and ***y*** fragments without (0 tat), with one (1 tat), and with two (2 tat) peptides attached; c) and d) level of occupancy of ***c*** (△, left axis) and ***y*** (▽, right axis) fragments with tat peptides as indicated.

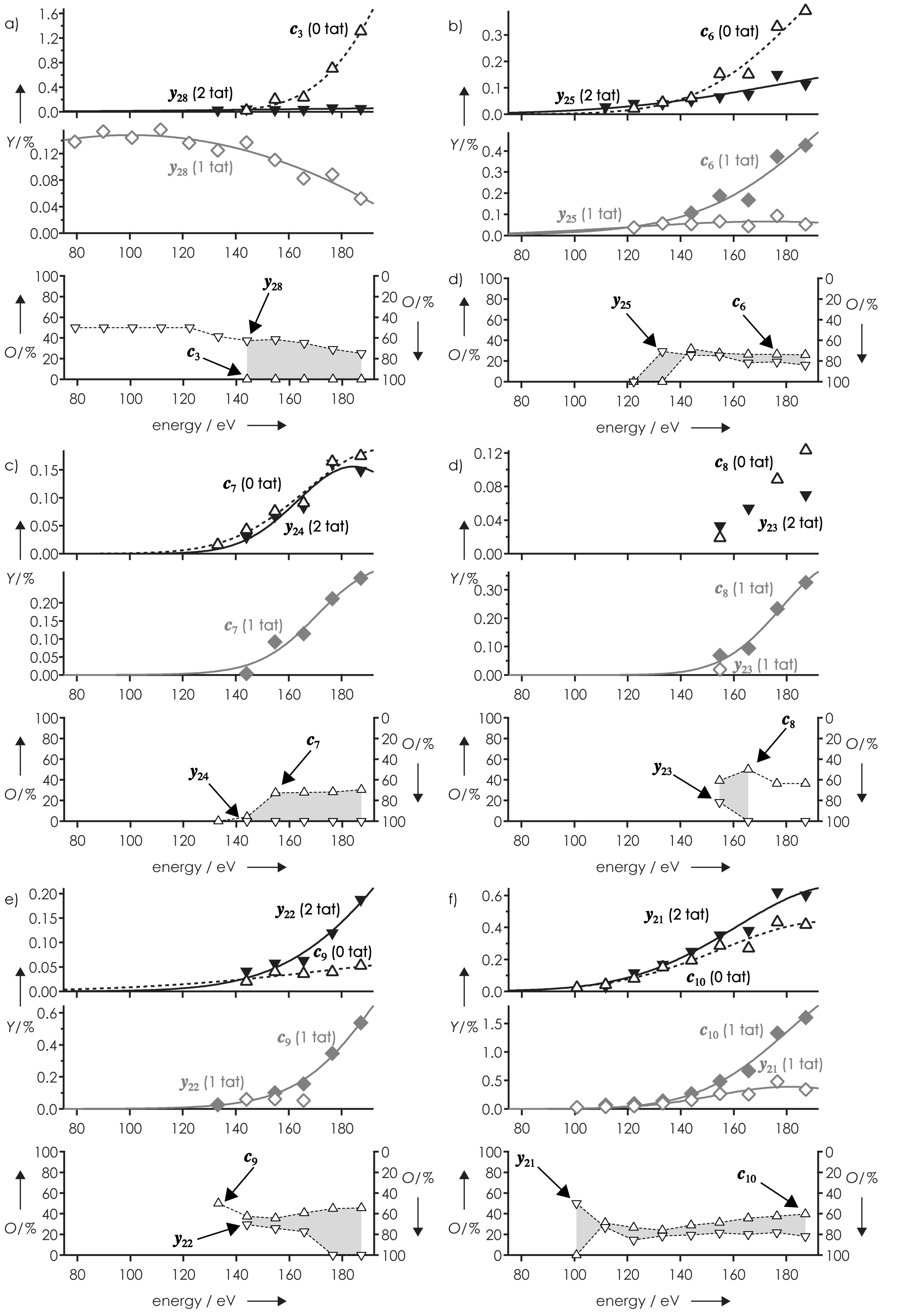

Figure S7. As in Figures 2c and 2d, yield of ***c*** and ***y*** ions from CAD of $(TAR+2tat-12H)^{12-}$, and level of occupancy with tat peptide vs. laboratory frame energy for cleavage at sites 3 (a) and 6-10 (b-f).

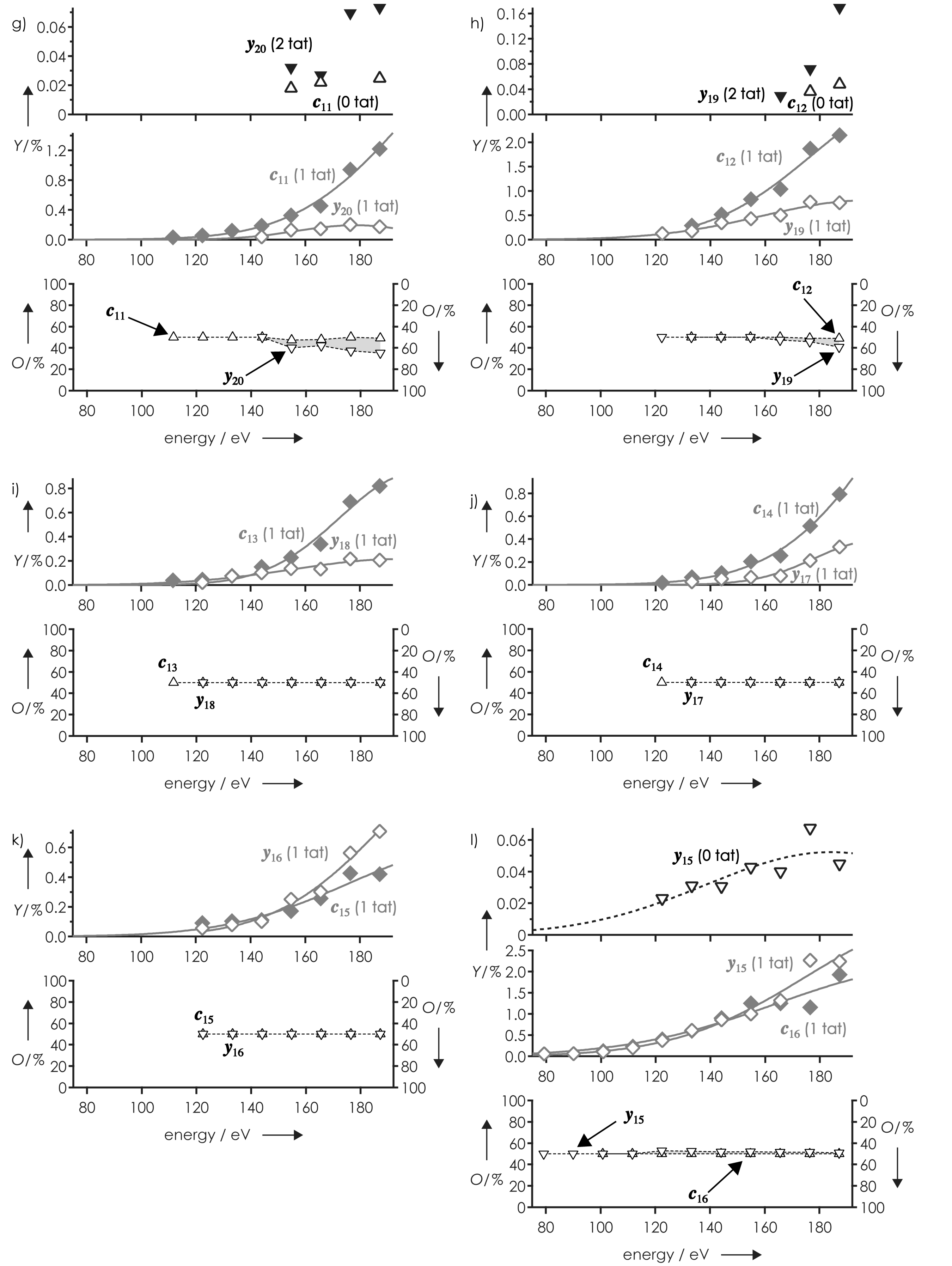

Figure S7 continued. yield of c and y ions from CAD of (TAR+2tat-12H)$^{12-}$, and level of occupancy with tat peptide vs. laboratory frame energy for cleavage at sites 11-16 (g-l).

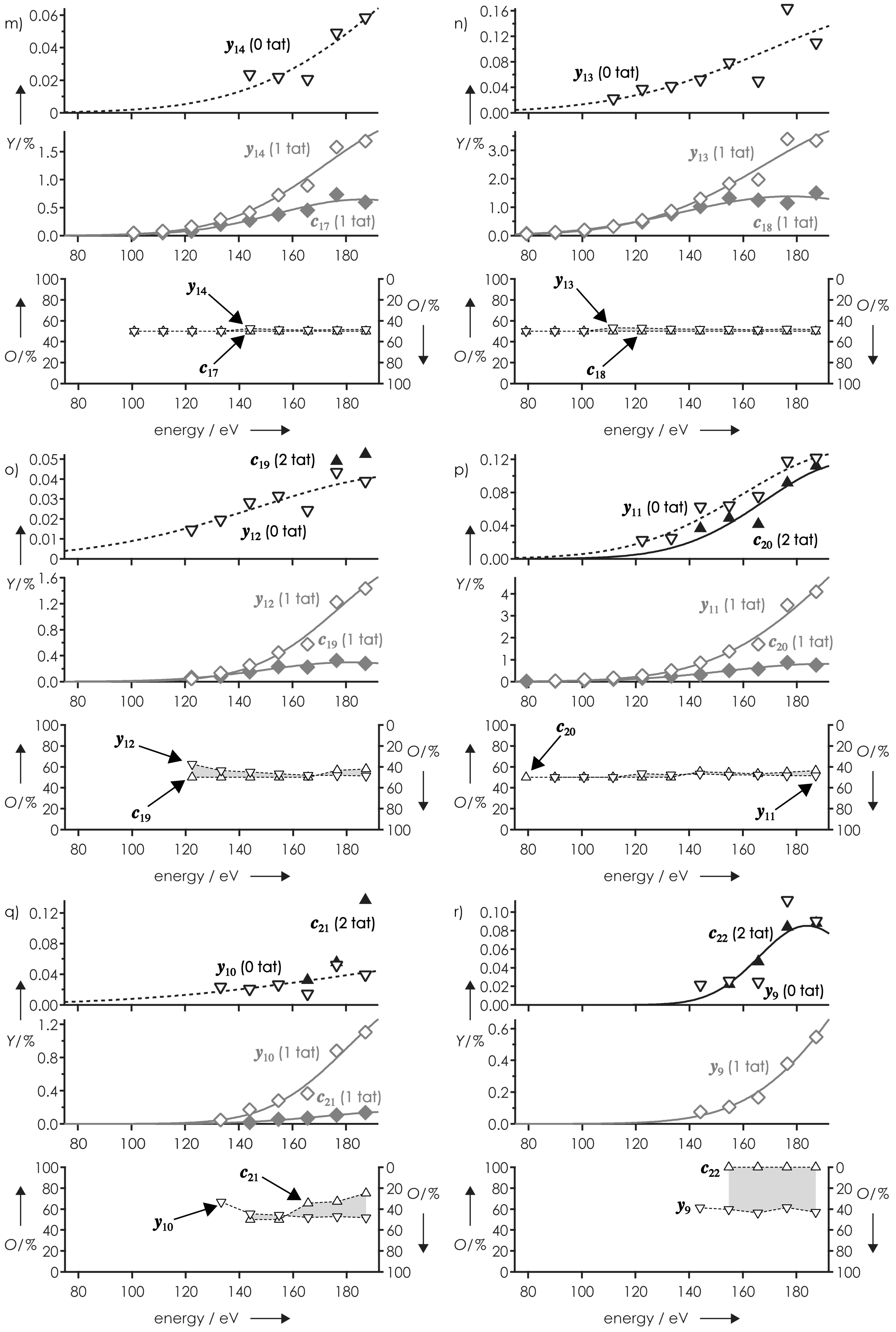

Figure S7 continued. yield of c and y ions from CAD of $(TAR+2tat-12H)^{12-}$, and level of occupancy with tat peptide vs. laboratory frame energy for cleavage at sites 17-22 (m-r).

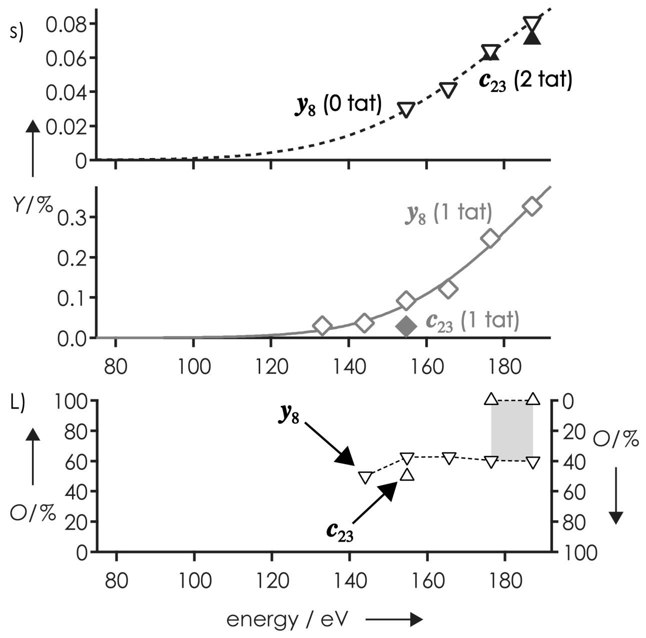

Figure S7 continued. yield of c and y ions from CAD of (TAR+2tat-12H)$^{12-}$, and level of occupancy with tat peptide vs. laboratory frame energy for cleavage at site 23 (s).

Table S1: Fragments from CAD of (TAR+tat-13H)$^{13-}$ ions at 120.9 eV laboratory frame energy, mass and m/z values refer to the monoisotopic peak.

m/z measured	z	m measured [Da]	m calculated [Da]	accuracy [ppm]	assignment
322.04475	1	323.05203	323.05185	-0.55	w_1
344.04022	1	345.04750	345.04743	-0.18	c_1
344.04022	2	690.09500	690.09487	-0.19	c_2
395.37062	3	1189.13369	1189.13417	0.41	c_4−C
432.38535	3	1300.17787	1300.17745	-0.32	c_4
438.38846	3	1318.18721	1318.18800	0.60	w_4
441.03908	2	884.09271	884.09289	0.20	c_3−C
445.57985	2	893.17426	893.17424	-0.01	y_3
456.57754	2	915.16963	915.16981	0.20	a_3
458.07209	1	459.07937	459.07913	-0.52	a_2−G
470.39599	3	1414.20980	1414.20913	-0.47	a_5−A
481.30642	4	1929.25479	1929.25459	-0.10	w_6
485.56304	2	973.14064	973.14057	-0.07	w_3
492.56202	4	1974.27719	1974.27739	0.11	c_6
496.56071	2	995.13598	995.13615	0.17	c_3
497.05125	3	1494.17557	1494.17546	-0.07	c_5−A
505.05497	3	1518.18674	1518.18669	-0.03	c_5−C
513.74177	3	1544.24714	1544.24698	-0.10	y_5
538.03831	1	539.04559	539.04545	-0.25	c_2−G
540.39726	3	1624.21361	1624.21330	-0.19	w_5
541.06141	4	2168.27475	2168.27541	0.31	c_7−A
542.06949	3	1629.23028	1629.22996	-0.20	c_5
542.57894	2	1087.17244	1087.17227	-0.16	y_4−G
546.05365	5	2735.30463	2735.30322	-0.52	w_9−C

547.11955	1	548.12683	548.12680	-0.04	y_2
553.57663	2	1109.16781	1109.16785	0.03	a_4–C
557.81279	4	2235.28025	2235.27989	-0.16	w_7
562.58204	2	1127.17863	1127.17841	-0.20	y_4–C
568.26202	5	2846.34646	2846.34648	0.00	w_9
573.54729	2	1149.10913	1149.12803	16.47	c_4–G
574.82525	4	2303.33009	2303.32991	-0.08	c_7
581.87196	5	2914.39618	2914.39650	0.11	c_9
593.23716	6	3565.46659	3565.46923	0.74	c_{11}
593.55991	2	1189.13438	1189.13417	-0.18	c_4–C
604.64554	7	4239.56972	4239.56919	-0.12	c_{13}
606.73537	3	1823.22794	1823.22799	0.03	c_6–G
609.09837	2	1220.21130	1220.21111	-0.16	a_4
612.06639	3	1839.22100	1839.22288	1.02	c_6–A
613.27687	5	3071.42073	3071.42143	0.23	y_{10}
614.08139	4	2460.35465	2460.35485	0.08	y_8
615.42229	3	1849.28869	1849.28827	-0.23	y_6
616.06611	5	3085.36692	3085.36730	0.13	c_{10}–A
617.56757	4	2474.29939	2474.30072	0.54	c_8–A
618.10365	2	1238.22185	1238.22167	-0.15	y_4
620.86819	5	3109.37735	3109.37852	0.38	c_{10}–C
623.24604	6	3745.51990	3745.52139	0.40	y_{12}
625.57066	6	3759.46761	3759.46726	-0.09	c_{12}–A
627.08589	1	628.09317	628.09314	-0.05	w_2
629.27024	5	3151.38758	3151.38776	0.06	w_{10}
634.07295	4	2540.32091	2540.32118	0.11	w_8
636.57407	6	3825.48807	3825.48771	-0.09	w_{12}
642.07736	3	1929.25391	1929.25459	0.35	w_6
643.07707	5	3220.42174	3220.42182	0.02	c_{10}
643.45384	8	5155.68893	5155.67706	-2.31	c_{16}
644.07210	7	4515.55564	4515.58259	5.98	w_{14}
648.07966	6	3894.52160	3894.52172	0.03	c_{12}
648.22174	7	4544.60312	4544.61047	1.62	c_{14}
649.08121	2	1300.17697	1300.17744	0.37	c_4
651.33177	4	2609.35617	2609.35521	-0.37	c_8
652.07653	5	3265.41903	3265.41947	0.13	y_{11}–G
655.57953	6	3939.52084	3939.51938	-0.37	y_{13}–G
657.08520	3	1974.27744	1974.27739	-0.03	c_6
658.08679	2	1318.18814	1318.18800	-0.11	w_4
658.24535	6	3955.51576	3955.51430	-0.37	y_{13}–A
660.07755	5	3305.42411	3305.42562	0.46	y_{11}–C
662.24683	6	3979.52463	3979.52558	0.24	y_{13}–C
664.51260	7	4658.63914	4658.64217	0.65	a_{15}–C
666.08137	7	4669.62053	4669.62043	-0.02	y_{15}–C
679.83915	4	2723.38569	2723.38690	0.45	a_9–C
680.75419	6	4090.56879	4090.56884	0.01	y_{13}
681.94454	7	4780.66272	4780.66369	0.20	y_{15}
682.28646	5	3416.46866	3416.46887	0.06	y_{11}
682.95490	8	5471.69745	5471.69660	-0.16	w_{17}
685.07576	5	3430.41518	3430.41472	-0.13	c_{11}–A
686.58312	8	5500.72313	5500.72450	0.25	c_{17}
686.86245	9	6190.82754	6190.81937	-1.32	c_{19}
689.08754	1	690.09482	690.09486	0.06	c_2
690.58774	4	2766.38005	2766.38013	0.03	y_9
691.80001	7	4849.65099	4849.65175	0.16	c_{15}
693.36881	7	4860.63261	4860.63002	-0.53	w_{15}
693.82879	4	2779.34427	2779.34201	-0.81	c_9–A
694.08263	6	4170.53947	4170.53515	-1.04	w_{13}
695.09592	3	2088.30960	2088.30909	-0.24	a_7–A

698.27987	5	3496.43575	3496.43520	-0.16	w_{11}
699.83145	4	2803.35491	2803.35325	-0.59	c_{9}–C
705.58718	6	4239.56671	4239.56918	0.58	c_{13}
706.09740	2	1414.20936	1414.20913	-0.16	a_{5}–A
706.86691	9	6370.86772	6370.87152	0.60	y_{20}
707.60009	4	2834.42948	2834.43017	0.24	a_{9}
708.41645	9	6384.81354	6384.81739	0.60	c_{20}–A
710.57941	4	2846.34674	2846.34648	-0.09	w_{9}
711.08921	8	5696.77189	5696.77157	-0.06	y_{18}
712.08656	5	3565.46917	3565.46922	0.01	c_{11}
712.83242	8	5710.71756	5710.71744	-0.02	c_{18}–A
715.75194	6	4300.55530	4300.56176	1.50	y_{14}–A
715.83470	8	5734.73581	5734.72867	-1.25	c_{18}–C
716.22450	7	5020.62241	5020.62255	0.03	c_{16}–A
717.43066	3	2155.31382	2155.31356	-0.12	y_{7}
719.65444	7	5044.63202	5044.63381	0.36	c_{16}–C
719.75489	6	4324.57302	4324.57298	-0.01	y_{14}–C
721.08429	8	5776.73256	5776.73789	0.92	w_{18}
721.75115	3	2168.27528	2168.27542	0.06	c_{7}–A
723.42289	9	6519.87150	6519.87183	0.05	c_{20}
724.59259	6	4353.59918	4353.60088	0.39	a_{14}–C
725.66247	7	5086.68822	5086.68897	0.15	y_{16}
727.59193	4	2914.39684	2914.39649	-0.12	c_{9}
729.71425	8	5845.77219	5845.77195	-0.04	c_{18}
734.89300	5	3679.50137	3679.50093	-0.12	a_{12}–A
735.33898	8	5890.77005	5890.76955	-0.09	y_{19}–G
735.51793	7	5155.67646	5155.67705	0.12	c_{16}
737.08656	7	5166.65685	5166.65532	-0.30	w_{16}
738.08260	2	1478.17975	1478.18055	0.54	c_{5}–G
738.26203	6	4435.61582	4435.61624	0.09	y_{14}
740.11371	3	2223.36296	2223.36358	0.28	a_{7}
743.10026	6	4464.64521	4464.64414	-0.24	a_{14}
744.08607	3	2235.28004	2235.27989	-0.07	w_{7}
745.20609	9	6715.92028	6715.91892	-0.20	y_{21}
746.08058	2	1494.17572	1494.17547	-0.17	c_{5}–A
748.09708	5	3745.52178	3745.52137	-0.11	y_{12}
750.88621	5	3759.46743	3759.46727	-0.04	c_{12}–A
751.58973	6	4515.58202	4515.58259	0.12	w_{14}
754.22001	8	6041.81832	6041.81899	0.11	y_{19}
756.42829	6	4544.61340	4544.61044	-0.65	c_{14}
761.76191	9	6864.92268	6864.91932	-0.49	c_{21}
764.09031	5	3825.48792	3825.48772	-0.05	w_{12}
765.51680	7	5365.66857	5365.66998	0.26	c_{17}–A
766.33681	4	3069.37635	3069.37239	-1.29	c_{10}–G
766.77006	3	2303.33200	2303.32990	-0.91	c_{7}
766.84815	4	3071.42169	3071.42141	-0.09	y_{10}
769.24017	7	5391.73214	5391.73028	-0.35	y_{17}
770.33455	4	3085.36732	3085.36729	-0.01	c_{10}–A
770.59511	6	4629.61432	4629.61430	0.00	y_{15}–G
771.11632	2	1544.24720	1544.24697	-0.15	y_{5}
772.84501	8	6190.81833	6190.81939	0.17	c_{19}
773.25910	6	4645.59826	4645.60922	2.36	y_{15}–A
773.62443	2	1549.26340	1549.26363	0.14	a_{5}
775.43209	6	4658.63618	4658.64217	1.29	a_{15}–C
776.09081	4	3108.39236	3108.39453	0.70	c_{10}–U
777.26180	6	4669.61446	4669.62043	1.28	y_{15}–C
777.89717	5	3894.52224	3894.52175	-0.12	c_{12}
780.66378	7	5471.69740	5471.69661	-0.14	w_{17}
784.10691	4	3140.45675	3140.45547	-0.41	a_{10}

784.81084	7	5500.72680	5500.72448	-0.42	c_{17}
786.83972	4	3151.38797	3151.38777	-0.06	w_{10}
787.09776	8	6304.84029	6304.85106	1.71	a_{20}–A
793.94351	6	4769.70472	4769.68543	-4.05	a_{15}
795.19912	10	7962.06395	7962.06336	-0.07	y_{25}
795.35192	8	6370.87357	6370.87152	-0.32	y_{20}
795.76993	6	4780.66322	4780.66370	0.10	y_{15}
797.09453	8	6384.81445	6384.81739	0.46	c_{20}–A
803.11985	1	804.12713	804.12656	-0.71	a_3–C
804.09836	4	3220.42254	3220.42180	-0.23	c_{10}
807.26877	6	4849.65628	4849.65176	-0.93	c_{15}
811.09947	2	1624.21349	1624.21331	-0.12	w_5
812.81708	7	5696.77050	5696.77157	0.19	y_{18}
813.60801	2	1629.23057	1629.22996	-0.37	c_5
814.80929	7	5710.71594	5710.71741	0.26	c_{18}–A
817.10646	5	4090.56866	4090.56882	0.04	y_{13}
819.11121	3	2460.35545	2460.35485	-0.25	y_8
824.23847	7	5776.72023	5776.73790	3.06	w_{18}
825.34788	4	3305.42063	3305.42561	1.51	y_{11}–C
832.60652	4	3334.45519	3334.45349	-0.51	a_{11}–G
834.10292	7	5845.77135	5845.77193	0.10	c_{18}
835.76384	6	5020.62670	5020.62257	-0.82	c_{16}–A
838.48314	8	6715.92333	6715.91895	-0.65	y_{21}
842.12220	3	2529.38843	2529.38888	0.18	a_8
846.77296	6	5086.68142	5086.68899	1.49	y_{16}
850.39172	7	5959.79298	5959.80363	1.79	a_{19}–G
853.10971	4	3416.46794	3416.46887	0.27	y_{11}
858.27222	6	5155.67695	5155.67706	0.02	c_{16}
862.10756	7	6041.80388	6041.81900	2.51	y_{19}
863.92343	12	10379.16848	10379.16388	-0.44	y_{28}+*tat*–G
865.25613	12	10395.16088	10395.15880	-0.20	y_{28}+*tat*–A
866.85884	13	11282.25951	11282.26395	0.39	c_{30}+*tat*
876.51083	12	10530.21728	10530.21331	-0.38	y_{28}+*tat*
883.42446	9	7959.88561	7959.88020	-0.68	y_{20}+*tat*
890.59106	10	8915.98336	8915.99425	1.22	y_{23}+*tat*
892.16761	1	893.17489	893.17424	-0.72	y_3
892.30646	9	8039.82364	8039.84656	2.85	w_{20}+*tat*
898.59053	10	8995.97805	8995.96059	-1.94	w_{23}+*tat*
898.64069	11	9896.12763	9896.11951	-0.82	y_{26}+*tat*
899.98015	9	8108.88680	8108.88059	-0.77	c_{20}+*tat*
905.49122	10	9064.98496	9064.99462	1.07	c_{23}+*tat*
905.90940	11	9976.08345	9976.08584	0.24	w_{26}+*tat*
906.36562	11	9981.10186	9981.10249	0.06	c_{26}+*tat*
913.76214	12	10977.23300	10977.22264	-0.94	c_{29}+*tat*
921.19469	10	9222.01966	9222.01955	-0.01	y_{24}+*tat*
921.76289	9	8304.93151	8304.92766	-0.46	y_{21}+*tat*
921.90785	11	10152.06639	10152.07330	0.68	c_{27}+*tat*–A
923.63733	2	1849.28921	1849.28826	-0.51	y_6
924.09140	11	10176.08548	10176.08453	-0.09	c_{27}+*tat*–C
926.59341	12	11131.20824	11131.21454	0.57	c_{30}+*tat*–G
927.92717	12	11147.21336	11147.20945	-0.35	c_{30}+*tat*–A
928.34081	8	7434.78468	7434.78064	-0.54	c_{18}+*tat*
928.55423	11	10225.17661	10225.17203	-0.45	y_{27}+*tat*
929.19164	10	9301.98916	9301.98589	-0.35	w_{24}+*tat*
930.64738	9	8384.89191	8384.89400	0.25	w_{21}+*tat*
934.18638	11	10287.13027	10287.12777	-0.24	c_{27}+*tat*
935.99856	10	9370.05836	9370.03590	-2.40	c_{24}+*tat*
938.31858	9	8453.93271	8453.92803	-0.55	c_{21}+*tat*
939.18107	12	11282.26016	11282.26396	0.34	c_{30}+*tat*

942.59673	8	7548.83208	7548.81234	-2.62	a_{19}+*tat*−G
944.09752	11	10396.15280	10396.16610	1.28	y_{28}+*tat*−A
945.58704	6	5679.56590	5679.57753	2.05	y_{13}+*tat*
952.66383	7	6675.69775	6675.69770	-0.01	y_{16}+*tat*
952.84681	8	7630.83273	7630.82771	-0.66	y_{19}+*tat*
954.10047	10	9551.07745	9551.07204	-0.57	y_{25}+*tat*
955.76481	9	8610.94879	8610.95298	0.49	y_{22}+*tat*
956.28445	11	10530.20899	10530.21325	0.40	y_{28}+*tat*
962.51961	7	6744.68824	6744.68573	-0.37	c_{16}+*tat*
962.84206	8	7710.79472	7710.79404	-0.09	w_{19}+*tat*
964.08861	7	6755.67120	6755.66403	-1.06	w_{16}+*tat*
964.65132	9	8690.92735	8690.91930	-0.93	w_{22}+*tat*
965.55487	11	10632.18365	10632.17522	-0.79	c_{28}+*tat*
966.59946	10	9676.06739	9676.06124	-0.64	c_{25}+*tat*
970.42185	6	5828.57476	5828.57790	0.54	c_{13}+*tat*
971.47218	8	7779.83565	7779.82808	-0.97	c_{19}+*tat*
972.20921	9	8758.94838	8758.96932	2.39	c_{22}+*tat*
973.49973	10	9745.07010	9745.07014	0.00	y_{26}+*tat*−G
975.09997	10	9761.07244	9761.06504	-0.76	y_{26}+*tat*−A
977.09704	8	7824.83453	7824.82571	-1.13	y_{20}+*tat*−A
977.50091	10	9785.08186	9785.07624	-0.58	y_{26}+*tat*−C
984.01592	11	10835.25513	10835.25458	-0.05	y_{29}+*tat*
985.72275	8	7893.84021	7893.85977	2.48	a_{20}+*tat*−A
986.13174	2	1974.27804	1974.27739	-0.33	c_6
988.60520	10	9896.12473	9896.11954	-0.53	y_{26}+*tat*
989.65835	9	8915.99065	8915.99421	0.40	y_{23}+*tat*
992.51832	7	6954.67915	6954.67873	-0.06	c_{17}+*tat*−A
993.97839	8	7959.88533	7959.88023	-0.64	y_{20}+*tat*
994.12984	1	995.13712	995.13615	-0.97	c_3
995.72156	8	7973.83069	7973.82611	-0.57	c_{20}+*tat*−A
996.24022	7	6980.73248	6980.73899	0.93	y_{17}+*tat*
996.60255	10	9976.09826	9976.08584	-1.25	w_{26}+*tat*
997.10153	10	9981.08806	9981.10244	1.44	c_{26}+*tat*
998.53838	9	8995.91091	8995.96059	5.53	w_{23}+*tat*
1000.08800	5	5005.47638	5005.47758	0.24	y_{11}+*tat*
1003.09718	6	6024.62671	6024.62498	-0.29	y_{14}+*tat*
1003.97397	8	8039.84997	8039.84656	-0.42	w_{20}+*tat*
1006.21436	9	9064.99473	9064.99462	-0.01	c_{23}+*tat*
1007.66504	7	7060.70624	7060.70533	-0.13	w_{17}+*tat*
1007.93697	6	6053.66548	6053.65285	-2.09	a_{14}+*tat*
1011.81200	7	7089.73492	7089.73323	-0.24	c_{17}+*tat*
1012.60295	8	8108.88183	8108.88059	-0.15	c_{20}+*tat*
1014.20046	10	10152.07737	10152.07334	-0.40	c_{27}+*tat*−A
1016.08194	5	5085.44608	5085.44391	-0.43	w_{11}+*tat*
1016.42329	6	6104.58340	6104.59130	1.29	w_{14}+*tat*
1018.22941	8	8153.89346	8153.87827	-1.87	y_{21}+*tat*−G
1018.88481	9	9179.02878	9179.02631	-0.27	a_{24}+*tat*−C
1019.80874	10	10208.16012	10208.16146	0.13	a_{27}+*tat*
1020.22675	8	8169.87221	8169.87315	0.11	y_{21}+*tat*−A
1021.26263	6	6133.61942	6133.61918	-0.04	c_{14}+*tat*
1021.51041	10	10225.17686	10225.17203	-0.47	y_{27}+*tat*
1023.22989	8	8193.89733	8193.88443	-1.58	y_{21}+*tat*−C
1023.66194	9	9222.02293	9222.01950	-0.37	y_{24}+*tat*
1026.85673	8	8222.91209	8222.91229	0.02	a_{21}+*tat*−G
1027.70544	10	10287.12714	10287.12779	0.06	c_{27}+*tat*
1029.88891	5	5154.48094	5154.47794	-0.58	c_{11}+*tat*
1031.22306	9	9290.07304	9290.06957	-0.37	a_{24}+*tat*
1032.54652	9	9301.98417	9301.98589	0.19	w_{24}+*tat*
1037.10904	8	8304.93050	8304.92763	-0.35	y_{21}+*tat*

1039.81935	7	7285.78636	7285.78027	-0.84	y_{18}+*tat*
1041.81096	7	7299.72766	7299.72616	-0.20	c_{18}+*tat*−A
1043.14847	2	2088.31149	2088.30909	-1.15	a_{7}−A
1045.24314	7	7323.75288	7323.73735	-2.12	c_{18}+*tat*−C
1047.10526	8	8384.90029	8384.89400	-0.75	w_{21}+*tat*
1051.24225	7	7365.74669	7365.74661	-0.01	w_{18}+*tat*
1055.73319	8	8453.92373	8453.92803	0.51	c_{21}+*tat*
1058.77496	6	6358.69341	6358.69414	0.11	a_{15}+*tat*
1060.60540	6	6369.67607	6369.67238	-0.58	y_{15}+*tat*
1061.10499	7	7434.78584	7434.78066	-0.70	c_{18}+*tat*
1065.89903	5	5334.53154	5334.53012	-0.27	y_{12}+*tat*
1069.98852	8	8567.96639	8567.95972	-0.78	a_{22}+*tat*−C
1072.10314	6	6438.66248	6438.66044	-0.32	c_{15}+*tat*
1075.36280	8	8610.96061	8610.95297	-0.89	y_{22}+*tat*
1081.89252	5	5414.49899	5414.49643	-0.47	w_{12}+*tat*
1083.86721	8	8678.99588	8679.00299	0.82	a_{22}+*tat*
1089.11145	7	7630.83107	7630.82770	-0.44	y_{19}+*tat*
1095.69970	5	5483.53487	5483.53047	-0.80	c_{12}+*tat*
1098.97401	7	7699.86897	7699.86175	-0.94	a_{19}+*tat*
1100.53454	7	7710.79271	7710.79405	0.17	w_{19}+*tat*
1100.59634	6	6609.62170	6609.63128	1.45	c_{16}+*tat*−A
1104.59908	6	6633.63814	6633.64250	0.66	c_{16}+*tat*−C
1110.39732	7	7779.83218	7779.82809	-0.52	c_{19}+*tat*
1111.61012	6	6675.70437	6675.69770	-1.00	y_{16}+*tat*
1123.10756	6	6744.68900	6744.68577	-0.48	c_{16}+*tat*
1124.93753	6	6755.66885	6755.66404	-0.71	w_{16}+*tat*
1134.90908	5	5679.58177	5679.57752	-0.75	y_{13}+*tat*
1136.11877	7	7959.88232	7959.88023	-0.26	y_{20}+*tat*
1138.10958	7	7973.81800	7973.82610	1.02	c_{20}+*tat*−A
1142.11268	6	6858.71974	6858.71747	-0.33	a_{17}+*tat*−G
1158.10458	6	6954.67114	6954.67871	1.09	c_{17}+*tat*−A
1162.44964	6	6980.74148	6980.73896	-0.36	y_{17}+*tat*
1164.10141	4	4660.43475	4660.43013	-0.99	y_{10}+*tat*
1164.70853	5	5828.57903	5828.57792	-0.19	c_{13}+*tat*
1180.61654	6	7089.74290	7089.73321	-1.37	c_{17}+*tat*
1186.75560	3	3563.28863	3563.28610	-0.71	c_{6}+*tat*
1187.51514	5	5942.61206	5942.60960	-0.42	a_{14}+*tat*−C
1201.35115	4	4809.43372	4809.43049	-0.67	c_{10}+*tat*
1203.91819	5	6024.62732	6024.62497	-0.39	y_{14}+*tat*
1209.72385	5	6053.65565	6053.65286	-0.46	a_{14}+*tat*
1213.29006	6	7285.78402	7285.78028	-0.51	y_{18}+*tat*
1225.71658	5	6133.61928	6133.61918	-0.02	c_{14}+*tat*
1238.12163	6	7434.77344	7434.78064	0.97	c_{18}+*tat*
1250.36267	4	5005.47979	5005.47757	-0.44	y_{11}+*tat*
1270.35333	4	5085.44243	5085.44391	0.29	w_{11}+*tat*
1272.92678	5	6369.67030	6369.67240	0.33	y_{15}+*tat*
1287.61367	4	5154.48379	5154.47794	-1.13	c_{11}+*tat*
1332.62636	4	5334.53454	5334.53010	-0.83	y_{12}+*tat*

Table S2: Fragments from CAD of $(TAR+2tat-12H)^{12-}$ ions at 154.8 eV laboratory frame energy, mass and *m/z* values refer to the monoisotopic peak.

m/z measured	*z*	*m* measured [Da]	*m* calculated [Da]	accuracy [ppm]	assignment
344.04055	1	345.04783	345.04743	-1.14	c_{1}
445.58001	2	893.17457	893.17424	-0.37	y_{3}
458.07202	1	459.07930	459.07913	-0.37	a_{2}−G
485.56316	2	973.14087	973.14057	-0.31	w_{3}

496.56103	2	995.13662	995.13615	-0.48	c_{3}
497.05149	3	1494.17631	1494.17546	-0.57	c_{5}–A
513.74199	3	1544.24781	1544.24697	-0.54	y_{5}
538.03851	1	539.04579	539.04545	-0.62	c_{2}–G
540.39718	3	1624.21337	1624.21330	-0.04	w_{5}
542.06957	3	1629.23054	1629.22996	-0.35	c_{5}
542.57735	2	1087.16925	1087.17226	2.77	y_{4}–G
547.11969	1	548.12697	548.12680	-0.30	y_{2}
553.57679	2	1109.16813	1109.16785	-0.26	a_{4}–C
557.81299	4	2235.28105	2235.27989	-0.52	w_{7}
562.58145	2	1127.17745	1127.17841	0.85	y_{4}–C
568.26245	5	2846.34863	2846.34648	-0.76	w_{9}
574.82481	4	2303.32835	2303.32991	0.68	c_{7}
593.55939	2	1189.13333	1189.13417	0.71	c_{4}–C
609.09846	2	1220.21147	1220.21111	-0.30	a_{4}
615.42239	3	1849.28899	1849.28827	-0.39	y_{6}
618.10368	2	1238.22192	1238.22167	-0.20	y_{4}
627.08608	1	628.09336	628.09314	-0.35	w_{2}
629.26975	5	3151.38513	3151.38776	0.84	w_{10}
634.07342	4	2540.32279	2540.32118	-0.63	w_{8}
642.07791	3	1929.25556	1929.25459	-0.50	w_{6}
643.07715	5	3220.42214	3220.42180	-0.10	c_{10}
649.08153	2	1300.17761	1300.17745	-0.13	c_{4}
651.33158	4	2609.35541	2609.35521	-0.07	c_{8}
657.08525	3	1974.27757	1974.27739	-0.09	c_{6}
658.08674	2	1318.18803	1318.18800	-0.02	w_{4}
680.75602	6	4090.57978	4090.56882	-2.68	y_{13}
682.28599	5	3416.46631	3416.46887	0.75	y_{11}
689.08761	1	690.09489	690.09486	-0.04	c_{2}
690.58769	4	2766.37986	2766.38013	0.10	y_{9}
695.09579	3	2088.30919	2088.30909	-0.05	a_{7}–A
698.28165	5	3496.44463	3496.43520	-2.70	w_{11}
705.58800	6	4239.57166	4239.56919	-0.58	c_{13}
706.09729	2	1414.20914	1414.20913	-0.01	a_{5}–A
707.60011	4	2834.42955	2834.43017	0.22	a_{9}
710.57941	4	2846.34676	2846.34648	-0.10	w_{9}
712.08649	5	3565.46882	3565.46922	0.11	c_{11}
717.43076	3	2155.31411	2155.31356	-0.26	y_{7}
721.75127	3	2168.27565	2168.27542	-0.10	c_{7}–A
727.59154	4	2914.39525	2914.39649	0.43	c_{9}
738.08250	2	1478.17955	1478.18055	0.68	c_{5}–G
738.26242	6	4435.61818	4435.61626	-0.43	y_{14}
744.08598	3	2235.27977	2235.27989	0.05	w_{7}
746.08029	2	1494.17513	1494.17547	0.23	c_{5}–A
748.09665	5	3745.51963	3745.52137	0.46	y_{12}
750.88398	5	3759.45628	3759.46726	2.92	c_{12}–A
758.08529	2	1518.18513	1518.18669	1.03	c_{5}–C
766.77001	3	2303.33187	2303.32990	-0.86	c_{7}
766.84846	4	3071.42296	3071.42141	-0.50	y_{10}
770.33410	4	3085.36551	3085.36729	0.58	c_{10}–A
771.11614	2	1544.24683	1544.24697	0.09	y_{5}
776.33492	4	3109.36879	3109.37854	3.14	c_{10}–C
777.89648	5	3894.51878	3894.52175	0.76	c_{12}
795.76988	6	4780.66296	4780.66370	0.15	y_{15}
803.11938	1	804.12666	804.12656	-0.12	a_{3}–C
804.09808	4	3220.42144	3220.42181	0.12	c_{10}
811.09923	2	1624.21301	1624.21331	0.19	w_{5}
813.60781	2	1629.23017	1629.22997	-0.12	c_{5}
817.10663	5	4090.56953	4090.56882	-0.18	y_{13}

819.11047	3	2460.35324	2460.35485	0.66	y_8
832.60706	4	3334.45735	3334.45349	-1.16	a_{11}–G
842.12274	3	2529.39005	2529.38888	-0.46	a_8
853.10940	4	3416.46669	3416.46885	0.63	y_{11}
858.27248	6	5155.67854	5155.67706	-0.29	c_{16}
868.77761	3	2609.35466	2609.35521	0.21	c_8
870.62342	2	1743.26139	1743.26165	0.15	a_6–G
883.08393	1	884.09121	884.09289	1.91	c_3–C
886.11484	5	4435.61058	4435.61626	1.28	y_{14}
892.16647	1	893.17375	893.17424	0.55	y_3
901.41315	6	5414.52256	5414.49643	-4.83	w_{12}+*tat*
906.78787	3	2723.38544	2723.38691	0.54	a_9–C
914.16251	1	915.16979	915.16982	0.04	a_3
923.63583	2	1849.28621	1849.28826	1.11	y_6
928.33798	8	7434.76205	7434.78064	2.50	c_{18}+*tat*
938.31910	9	8453.93739	8453.92803	-1.11	c_{21}+*tat*
942.50820	7	6604.60834	6604.61463	0.95	w_{16}+*tat*–G
944.78959	7	6620.57807	6620.60954	4.76	w_{16}+*tat*–A
945.59075	6	5679.58816	5679.57753	-1.87	y_{13}+*tat*
946.14864	2	1894.31183	1894.31106	-0.41	a_6
952.66318	7	6675.69322	6675.69770	0.67	y_{16}+*tat*
952.84440	8	7630.81341	7630.82771	1.88	y_{19}+*tat*
954.58702	8	7644.75437	7644.77358	2.52	c_{19}+*tat*–A
955.76386	9	8610.94023	8610.95297	1.48	y_{22}+*tat*
957.59017	8	7668.77957	7668.78481	0.68	c_{19}+*tat*–C
958.91751	6	5759.54872	5759.54387	-0.84	w_{13}+*tat*
962.51923	7	6744.68557	6744.68577	0.03	c_{16}+*tat*
962.84191	8	7710.79349	7710.79404	0.07	w_{19}+*tat*
964.08728	7	6755.66187	6755.66403	0.32	w_{16}+*tat*
970.42187	6	5828.57489	5828.57790	0.52	c_{13}+*tat*
971.47023	8	7779.82008	7779.82811	1.03	c_{19}+*tat*
972.21074	9	8758.96211	8758.96932	0.82	c_{22}+*tat*
978.80376	7	6858.67726	6858.71747	5.87	a_{17}+*tat*–G
985.72422	8	7893.85198	7893.85977	0.99	a_{20}+*tat*–A
986.08230	7	6909.62704	6909.65591	4.18	w_{17}+*tat*–G
986.13197	2	1974.27849	1974.27739	-0.56	c_6
988.37322	7	6925.66348	6925.65082	-1.83	w_{17}+*tat*–A
989.42790	6	5942.61106	5942.60960	-0.25	a_{14}+*tat*–C
989.65819	9	8915.98920	8915.99425	0.57	y_{23}+*tat*
991.80101	7	6949.65801	6949.66206	0.58	w_{17}+*tat*–C
992.51656	7	6954.66686	6954.67871	1.71	c_{17}+*tat*–A
993.97786	8	7959.88113	7959.88019	-0.12	y_{20}+*tat*
994.12800	1	995.13528	995.13615	0.88	c_3
995.72044	8	7973.82175	7973.82611	0.55	c_{20}+*tat*–A
996.24072	7	6980.73598	6980.73899	0.43	y_{17}+*tat*
998.72119	8	7997.82770	7997.83731	1.20	c_{20}+*tat*–C
1000.08678	5	5005.47028	5005.47758	1.46	y_{11}+*tat*
1003.09636	6	6024.62182	6024.62497	0.52	y_{14}+*tat*
1006.21461	9	9064.99702	9064.99461	-0.27	c_{23}+*tat*
1007.66497	7	7060.70570	7060.70533	-0.05	w_{17}+*tat*
1011.81084	7	7089.72682	7089.73323	0.91	c_{17}+*tat*
1012.60193	8	8108.87364	8108.88059	0.86	c_{20}+*tat*
1016.08116	5	5085.44218	5085.44391	0.34	w_{11}+*tat*
1016.42406	6	6104.58801	6104.59130	0.54	w_{14}+*tat*
1021.26220	6	6133.61687	6133.61918	0.38	c_{14}+*tat*
1023.23038	8	8193.90125	8193.88440	-2.06	y_{21}+*tat*–C
1023.66198	9	9222.02328	9222.01950	-0.41	y_{24}+*tat*
1029.88835	5	5154.47815	5154.47794	-0.04	c_{11}+*tat*
1030.13001	5	5155.68643	5155.67706	-1.82	c_{16}

1035.42691	6	6218.60512	6218.62299	2.88	y_{15}+*tat*−G
1037.10850	8	8304.92622	8304.92763	0.17	y_{21}+*tat*
1038.09628	6	6234.62134	6234.61790	-0.55	y_{15}+*tat*−A
1039.81764	7	7285.77442	7285.78027	0.80	y_{18}+*tat*
1040.26721	6	6247.64689	6247.65088	0.64	a_{15}+*tat*−C
1041.80974	7	7299.71915	7299.72616	0.96	c_{18}+*tat*−A
1045.24054	7	7323.73471	7323.73735	0.36	c_{18}+*tat*−C
1051.24173	7	7365.74304	7365.74661	0.48	w_{18}+*tat*
1052.01323	10	10530.20510	10530.21334	0.78	y_{28}+*tat*
1052.69542	5	5268.51348	5268.51396	0.09	a_{12}+*tat*−A
1055.73254	8	8453.91855	8453.92803	1.12	c_{21}+*tat*
1058.77451	6	6358.69071	6358.69414	0.54	a_{15}+*tat*
1060.22199	9	9551.06340	9551.07210	0.91	y_{25}+*tat*
1060.60466	6	6369.67163	6369.67238	0.12	y_{15}+*tat*
1061.10391	7	7434.77831	7434.78064	0.31	c_{18}+*tat*
1065.89801	5	5334.52641	5334.53010	0.69	y_{12}+*tat*
1067.52643	7	7479.73595	7479.77830	5.67	y_{19}+*tat*−G
1071.59654	12	12871.24580	12871.27264	2.09	c_{30}+2 *tat*
1072.09870	6	6438.63586	6438.66047	3.83	c_{15}+*tat*
1073.92847	6	6449.61448	6449.63874	3.76	w_{15}+*tat*
1075.36166	8	8610.95151	8610.95299	0.17	y_{22}+*tat*
1077.39720	7	7548.83134	7548.81234	-2.52	a_{19}+*tat*−G
1081.89257	5	5414.49926	5414.49643	-0.52	w_{12}+*tat*
1089.10970	7	7630.81885	7630.82770	1.16	y_{19}+*tat*
1095.69828	5	5483.52780	5483.53047	0.49	c_{12}+*tat*
1100.53090	7	7710.76720	7710.79405	3.48	w_{19}+*tat*
1100.59537	6	6609.61588	6609.63128	2.33	c_{16}+*tat*−A
1104.59968	6	6633.64171	6633.64250	0.12	c_{16}+*tat*−C
1107.89486	5	5544.51066	5544.52302	2.23	y_{13}+*tat*−A
1110.00487	11	12221.13358	12221.18396	4.13	c_{28}+2 *tat*
1110.39339	7	7779.80467	7779.82808	3.01	c_{19}+*tat*
1111.60871	6	6675.69592	6675.69770	0.27	y_{16}+*tat*
1118.50243	5	5597.54853	5597.56216	2.44	a_{13}+*tat*−G
1123.10721	6	6744.68690	6744.68576	-0.17	c_{16}+*tat*
1124.84330	4	4503.40231	4503.40521	0.65	c_{9}+*tat*
1124.93612	6	6755.66039	6755.66404	0.54	w_{16}+*tat*
1126.68623	7	7893.85457	7893.85977	0.66	a_{20}+*tat*−A
1134.90768	5	5679.57480	5679.57752	0.48	y_{13}+*tat*
1136.11902	7	7959.88410	7959.88023	-0.49	y_{20}+*tat*
1138.10913	7	7973.81486	7973.82610	1.41	c_{20}+*tat*−A
1141.37737	11	12566.23106	12566.23141	0.03	c_{29}+2 *tat*
1142.10565	6	6858.67756	6858.71747	5.82	a_{17}+*tat*−G
1147.50281	10	11485.10086	11485.12824	2.39	y_{26}+2 *tat*
1150.90022	5	5759.53751	5759.54387	1.11	w_{13}+*tat*
1155.50151	10	11565.08783	11565.09455	0.58	w_{26}+2 *tat*
1156.00284	10	11570.10116	11570.11120	0.87	c_{26}+2 *tat*
1158.10424	6	6954.66907	6954.67868	1.38	c_{17}+*tat*−A
1159.83924	11	12769.31168	12769.31077	-0.07	y_{30}+2 *tat*
1162.44945	6	6980.74033	6980.73896	-0.20	y_{17}+*tat*
1164.10076	4	4660.43216	4660.43013	-0.44	y_{10}+*tat*
1164.70867	5	5828.57974	5828.57792	-0.31	c_{13}+*tat*
1169.10814	11	12871.26958	12871.27266	0.24	c_{30}+2 *tat*
1173.09923	10	11741.06510	11741.08204	1.44	c_{27}+2 *tat*−A
1175.50212	10	11765.09392	11765.09324	-0.06	c_{27}+2 *tat*−C
1175.77736	6	7060.70784	7060.70532	-0.36	w_{17}+*tat*
1180.40840	10	11814.15676	11814.18074	2.03	y_{27}+2 *tat*
1180.61210	6	7089.71627	7089.73321	2.39	c_{17}+*tat*
1182.76981	9	10653.99378	10654.00329	0.89	c_{23}+2 *tat*
1184.08894	4	4740.38487	4740.39648	2.45	w_{10}+*tat*

1185.41286	7	8304.94099	8304.92766	-1.61	y_{21}+*tat*
1186.60653	10	11876.13804	11876.13654	-0.13	c_{27}+*2 tat*
1186.75373	3	3563.28303	3563.28610	0.86	c_{6}+*tat*
1187.51509	5	5942.61183	5942.60960	-0.38	a_{14}+*tat*−C
1195.44036	9	10768.02873	10768.07396	4.20	a_{24}+*2 tat*−C
1199.61938	6	7203.75993	7203.76490	0.69	a_{18}+*tat*−G
1200.21803	9	10811.02777	10811.02827	0.05	y_{24}+*2 tat*
1201.35025	4	4809.43010	4809.43049	0.08	c_{10}+*tat*
1203.91671	5	6024.61995	6024.62497	0.83	y_{14}+*tat*
1209.10224	9	10890.98564	10890.99459	0.82	w_{24}+*2 tat*
1209.72279	5	6053.65035	6053.65286	0.41	a_{14}+*tat*
1210.91666	10	12119.23940	12119.22204	-1.43	y_{28}+*2 tat*
1211.23158	8	9697.91086	9697.88932	-2.22	c_{20}+*2 tat*
1213.29045	6	7285.78638	7285.78028	-0.84	y_{18}+*tat*
1215.61256	6	7299.71900	7299.72615	0.98	c_{18}+*tat*−A
1216.66386	9	10959.04020	10959.04461	0.40	c_{24}+*2 tat*
1218.90796	10	12199.15234	12199.18835	2.95	w_{28}+*2 tat*
1219.90955	5	6104.58411	6104.59130	1.18	w_{14}+*tat*
1221.10723	10	12221.14506	12221.18394	3.18	c_{28}+*2 tat*
1225.71647	5	6133.61873	6133.61917	0.07	c_{14}+*tat*
1229.85482	4	4923.44839	4923.46221	2.81	a_{11}+*tat*−G
1235.73405	8	9893.93058	9893.93635	0.58	y_{21}+*2 tat*
1236.77879	9	11140.07457	11140.08078	0.56	y_{25}+*2 tat*
1238.12267	6	7434.77969	7434.78062	0.12	c_{18}+*tat*
1241.41886	10	12424.26139	12424.26334	0.16	y_{29}+*2 tat*
1242.71353	5	6218.60402	6218.62297	3.05	y_{15}+*tat*−G
1248.52210	5	6247.64690	6247.65088	0.64	a_{15}+*tat*−C
1250.36159	4	5005.47547	5005.47757	0.42	y_{11}+*tat*
1250.66821	9	11265.07937	11265.06990	-0.84	c_{25}+*2 tat*
1254.36055	8	10042.94261	10042.93675	-0.58	c_{21}+*2 tat*
1257.12746	6	7548.80842	7548.81234	0.52	a_{19}+*tat*−G
1260.11067	9	11350.06149	11350.07373	1.08	y_{26}+*2 tat*−A
1263.33635	9	11379.09262	11379.14055	4.22	a_{26}+*2 tat*−C
1264.51520	5	6327.61236	6327.61722	0.77	c_{15}+*tat*−C
1270.35275	4	5085.44011	5085.44391	0.75	w_{11}+*tat*
1270.73118	5	6358.69231	6358.69414	0.29	a_{15}+*tat*
1272.92552	5	6369.66397	6369.67240	1.32	y_{15}+*tat*
1273.98740	8	10199.95742	10199.96171	0.42	y_{22}+*2 tat*
1275.11752	9	11485.12317	11485.12823	0.44	y_{26}+*2 tat*
1282.49646	8	10268.02991	10268.01170	-1.77	a_{22}+*2 tat*
1283.98209	8	10279.91491	10279.92801	1.28	w_{22}+*2 tat*
1284.56106	9	11570.11503	11570.11122	-0.33	c_{26}+*2 tat*
1286.72266	5	6438.64967	6438.66047	1.68	c_{15}+*tat*
1287.61049	4	5154.47105	5154.47793	1.34	c_{11}+*tat*
1292.48807	8	10347.96281	10347.97803	1.47	c_{22}+*2 tat*
1296.43855	3	3892.33747	3892.33861	0.29	c_{7}+*tat*
1298.85877	4	5199.46419	5199.47561	2.20	y_{12}+*tat*−A
1311.67800	9	11814.16746	11814.18075	1.13	y_{27}+*2 tat*
1312.11845	8	10505.00580	10505.00299	-0.27	y_{23}+*2 tat*
1316.11177	7	9219.83333	9219.83643	0.34	y_{19}+*2 tat*
1316.11997	4	5268.50897	5268.51396	0.95	a_{12}+*tat*−A
1322.11141	8	10584.94953	10584.96930	1.87	w_{23}+*2 tat*
1330.74642	8	10654.02957	10654.00333	-2.46	c_{23}+*2 tat*
1332.62557	4	5334.53139	5334.53009	-0.24	y_{12}+*tat*
1334.13204	5	6675.69657	6675.69770	0.17	y_{16}+*tat*
1336.10732	4	5348.45840	5348.47597	3.29	c_{12}+*tat*−A
1337.39636	7	9368.82543	9368.83677	1.21	c_{19}+*2 tat*
1344.99678	8	10768.03248	10768.07396	3.86	a_{24}+*2 tat*−C
1347.93045	5	6744.68864	6744.68577	-0.43	c_{16}+*tat*

1348.78150	3	4049.36631	4049.36356	-0.68	y_8+*tat*
1350.36805	8	10811.00261	10811.02826	2.37	y_{24}+*2 tat*
1352.61612	4	5414.49358	5414.49643	0.53	w_{12}+*tat*
1358.87585	8	10879.06502	10879.07828	1.22	a_{24}+*2 tat*
1360.36629	8	10890.98850	10890.99460	0.56	w_{24}+*2 tat*
1363.11853	7	9548.88064	9548.88894	0.87	y_{20}+*2 tat*
1369.87514	4	5483.52968	5483.53046	0.14	c_{12}+*tat*
1370.73317	5	6858.70223	6858.71747	2.22	a_{17}+*tat*–G
1384.40574	7	9697.89115	9697.88927	-0.19	c_{20}+*2 tat*
1387.94126	6	8333.69124	8333.69450	0.39	c_{16}+*2 tat*
1391.50113	8	11140.06725	11140.08078	1.22	y_{25}+*2 tat*
1398.38083	4	5597.55244	5597.56216	1.74	a_{13}+*tat*–G
1398.44604	3	4198.35995	4198.36393	0.95	c_8+*tat*
1400.69451	7	9811.91250	9811.92100	0.87	a_{21}+*2 tat*–G
1412.41089	7	9893.92719	9893.93637	0.93	y_{21}+*2 tat*
1414.40885	7	9907.91287	9907.88220	-3.10	c_{21}+*2 tat*–A
1416.93626	5	7089.71768	7089.73321	2.19	c_{17}+*tat*
1418.88552	4	5679.57120	5679.57753	1.12	y_{13}+*tat*
1433.69619	7	10042.92427	10042.93675	1.24	c_{21}+*2 tat*
1438.87357	4	5759.52339	5759.54387	3.56	w_{13}+*tat*
1445.44495	6	8678.71336	8678.74190	3.29	c_{17}+*2 tat*
1450.78967	3	4355.39083	4355.38885	-0.46	y_9+*tat*
1456.12799	7	10199.94685	10199.96168	1.46	y_{22}+*2 tat*
1456.13432	4	5828.56639	5828.57790	1.98	c_{13}+*tat*
1478.12015	6	8874.76456	8874.78896	2.75	y_{18}+*2 tat*
1484.64242	4	5942.59877	5942.60959	1.82	a_{14}+*tat*–C
1500.12915	3	4503.40929	4503.40522	-0.91	c_9+*tat*
1502.95721	6	9023.78694	9023.78936	0.27	c_{18}+*2 tat*
1505.14874	4	6024.62406	6024.62497	0.15	y_{14}+*tat*
1512.40431	4	6053.64635	6053.65286	1.08	a_{14}+*tat*
1535.62746	6	9219.80839	9219.83642	3.04	y_{19}+*2 tat*
1552.47058	3	4660.43358	4660.43014	-0.74	y_{10}+*tat*
1602.13307	3	4809.42103	4809.43051	1.97	c_{10}+*tat*
1667.48117	3	5005.46535	5005.47758	2.44	y_{11}+*tat*
1717.15226	3	5154.47861	5154.47794	-0.13	c_{11}+*tat*
